# Macrophage Polarization Modulates FcγR- and CD13-Mediated Phagocytosis and Reactive Oxygen Species Production, Independently of Receptor Membrane Expression

**DOI:** 10.3389/fimmu.2017.00303

**Published:** 2017-03-27

**Authors:** Elizabeth Mendoza-Coronel, Enrique Ortega

**Affiliations:** ^1^Departamento de Inmunología, Instituto de Investigaciones Biomédicas, Universidad Nacional Autónoma de México, Ciudad Universitaria, Mexico City, Mexico

**Keywords:** macrophage activation, cytokines, macrophage effector functions, M1–M2 macrophages, phagocytosis

## Abstract

In response to microenvironmental cues, macrophages undergo a profound phenotypic transformation acquiring distinct activation phenotypes ranging from pro-inflammatory (M1) to anti-inflammatory (M2). To study how activation phenotype influences phagocytosis and production of reactive oxygen species (ROS) mediated by receptors for IgG antibodies (Fcγ receptors) and by CD13, human monocyte-derived macrophages were polarized to distinct phenotypes using IFN-γ (Mϕ-IFN-γ), IL-4 (Mϕ-IL-4), or IL-10 (Mϕ-IL-10). Phenotypically, Mϕ-IFN-γ were characterized as CD14^+^CD80^+^CD86^+^ cells, Mϕ-IL-4 as CD209^high^CD206^+^CD11b^+^CD14^low^, and Mϕ-IL-10 as CD16^+^CD163^+^ cells. Compared to non-polarized macrophages, FcγRI expression increased in Mϕ-IFN-γ and Mϕ-IL-10 and FcγRIII expression increased in Mϕ-IL-10. None of the polarizing cytokines modified FcγRII or CD13 expression. Functionally, we found that cytokine-mediated activation significantly and distinctively affected FcγR- and CD13-mediated phagocytosis and ROS generation. Compared to non-polarized macrophages, FcγRI-, FcγRII-, and CD13-mediated phagocytosis was significantly increased in Mϕ-IL-10 and decreased in Mϕ-IFN-γ, although both cytokines significantly upregulated FcγRI expression. IL-10 also increased phagocytosis of *Escherichia coli*, showing that the effect of IL-10 on macrophage phagocytosis is not specific for a particular receptor. Interestingly, Mϕ-IL-4, which showed poor FcγR- and CD13-mediated phagocytosis, showed very high phagocytosis of *E. coli* and zymosan. Coupled with phagocytosis, macrophages produce ROS that contribute to microbial killing. As expected, Mϕ-IFN-γ showed significant production of ROS after FcγRI-, FcγRII-, or CD13-mediated phagocytosis. Unexpectedly, we found that Mϕ-IL-10 can also produce ROS after simultaneous stimulation through several phagocytic receptors, as coaggregation of FcγRI/FcγRII/CD13 induced a belated but significant ROS production. Together, these results demonstrate that activation of macrophages by each cytokine distinctly modulates expression of phagocytic receptors, FcγR- and CD13-mediated phagocytosis, and ROS production.

## Introduction

Macrophages are a phenotypically and functionally heterogeneous group of myeloid cells, with a high degree of plasticity. In tissues, macrophages respond to the local cytokine milieu with the acquisition of distinct functional phenotypes. In response to TLRs ligands and IFN-γ, macrophages undergo classical M1 activation, whereas they undergo alternative M2 activation after stimulation by IL-4/IL-13 or other stimuli. The M1-M2 model of macrophage polarization was proposed to reflect the Th1–Th2 polarization of T cells’ responses (1). The M1 phenotype is characterized by secretion of high levels of pro-inflammatory cytokines, high production of reactive nitrogen and oxygen intermediates, promotion of Th1 responses, and strong microbicidal and tumoricidal activity (2–5). On the other hand, M2 phenotype, originally designating the phenotype obtained by treatment of macrophages with IL-4, has been subdivided into various phenotypes: M2a (induced by IL-4 or IL-13), M2b (induced by immune complexes plus bacterial LPS), and M2c (induced by IL-10, glucocorticoids, and TGF-β) (2). M2a macrophages are characterized by the expression of mannose receptors and production of ornithine and polyamines through the arginase pathway, and are important in infections by parasites, allergy, and type II inflammation. M2c macrophages are characterized by high expression of scavenger receptors and higher production of IL-10, and are important in immunoregulatory functions and tissue remodeling (3, 5, 6).

Phagocytosis, endocytosis, secretion, and microbial killing are among the main functions of macrophages both in homeostasis and during microbial or damage-related threats (7). Phagocytosis is an essential function for the removal of dead or dying cells, tissue remodeling, and host defense. Phagocytosis is an actin-dependent process used by phagocytes to internalize particles greater than 0.5 μm in diameter (8–10). In monocytes and macrophages, phagocytosis can be mediated by a wide variety of phagocytic receptors, including receptors for IgG antibodies (FcγRs) and CD13. FcγRs are among the best characterized phagocytic receptors. Binding of IgG-opsonized particles to FcγRs on the surface of a phagocyte induces crosslinking of the receptors and triggers a series of cellular responses that are important for inflammation and immunity. These responses include phagocytosis, production of reactive oxygen species (ROS), antibody-dependent cell-mediated cytotoxicity, release of pro-inflammatory mediators, and production of cytokines (11, 12). For its part, CD13 is a membrane peptidase, which participates in a wide variety of functions (13). CD13 is highly expressed on myeloid cells, and we have recently shown that in human monocytes and macrophages, CD13 is a competent phagocytic receptor capable of mediating phagocytosis, independently of other receptors (14). CD13 can also modulate phagocytosis mediated by FcγRs (15) and by other receptors (16). In addition, CD13 crosslinking induces ROS production in macrophages (14).

Cytokine-mediated activation of macrophages is known to regulate the expression of many different membrane receptors, including the different classes of FcγRs (17, 18). Usually, it is presumed that changes in the expression level of a given receptor results in corresponding changes in the magnitude of the responses mediated by the receptor. However, we (19) and others (20, 21) have reported a lack of correlation between changes in expression levels of FcγRs and phagocytosis or antibody-mediated cell cytotoxicity mediated by them. To test the hypothesis that the ability of a cell to perform specific receptor-mediated functions depends more on the polarization state of the cell than on the expression level of the receptor, we activated human macrophages to three distinct functional phenotypes and comparatively determined the expression levels of FcγRs and CD13, as well as phagocytosis and ROS production mediated by these receptors. Our results demonstrate that the polarization state of macrophages, more than changes in receptor expression, determines the cell’s capacity for phagocytosis and production of ROS.

## Materials and Methods

### Reagents and Antibodies

Fetal bovine serum (FBS), RPMI-1640 medium, sodium pyruvate solution, MEM non-essential amino acids solution, l-glutamine, penicillin, and streptomycin were purchased from Gibco Life Technologies (Carlsbad, CA, USA). Lymphoprep was from Axis-Shield PoC AS (Oslo, Norway). Recombinant human IFN-γ (rhIFN-γ), recombinant human IL-10 (rhIL-10), and recombinant human IL-4 (rhIL-4) were purchased from PeproTech (Rocky Hill, NJ, USA). Carboxy-H2DFFDA and carboxyfluorescein succinimidyl ester (CFSE) were from Molecular Probes by Life Technologies (Eugene, OR, USA). Sulfo-NHS-Biotin was from Thermo Scientific (Waltham, MA, USA); streptavidin was from Calbiochem (San Diego, CA, USA), and bovine serum albumin (BSA) was from Sigma (St. Louis, MO, USA). All culture media were supplemented with 10% heat-inactivated FBS and 1 mM sodium pyruvate, 0.1 mM non-essential amino acids solution, 0.1 mM l-glutamine, 100 U/mL penicillin, and 100 μg/mL streptomycin (complete media). Cultures were maintained in a humidified atmosphere at 37°C with 5% CO_2_. Murine monoclonal anti-hCD13 (clone 452, IgG1) was purified in our laboratory from culture supernatants of the hybridoma, kindly donated by Dr. Meenhard Herlyn (Wistar Institute of Anatomy and Biology, Philadelphia, PA, USA). Murine monoclonal IgG1 anti-human FcγRI (clone 32.2) and murine monoclonal IgG2a anti-human FcγRII (clone IV.3) mAbs were purified in our laboratory from supernatants of the corresponding hybridomas obtained from American Type Culture Collection. Fab fragments of the antibodies were prepared with immobilized ficin (Pierce, Rockford, IL, USA) following the manufacturer’s instructions.

Biotin-F(ab′)2 fragments of goat anti-mouse IgG (H + L) were from Zymed (Invitrogen) and from Life Technologies (Eugene, OR, USA). Goat anti-mouse-FITC, used as a secondary antibody for immunostaining, was from Zymed (Invitrogen). Monoclonal mouse anti-human CD14 (clone RM032, IgG2a) was from Beckman Coulter Company (CA, USA). Monoclonal mouse anti-human CD80 (clone 2D10, IgG1) was from BioLegend (San Diego, CA, USA). Monoclonal mouse anti-human CD209 (clone DCN 47.5, IgG1) was from Miltenyi Biotec (Bergisch Gladbach, Germany). Monoclonal mouse anti-human CD11b (clone ICRF44, IgG1), CD11c (clone B-ly6, IgG1, κ), CD86 (clone 2331 [FUN-1], IgG1), CD206 (clone 19.2, IgG1), CD163 (clone GHI/61, IgG1, κ), and CD16 (clone 3G8, IgG1, κ) were all from BD Pharmingen (San Diego, CA, USA). Trizol, Turbo DNA-free kit, Oligo (dT) 12–18 primers and dNTP Mix 10 nM were from Invitrogen. Moloney Murine Leukemia Virus Reverse Transcriptase (M-MLV-RT) was from Promega. SYBR Green PCR Master Mix was from Applied Biosystems.

### Human Monocyte-Derived Macrophages (hMDMs) and *In Vitro* Polarization

Buffy coats from healthy male donors were obtained from the Central Blood Bank of the Centro Médico Nacional Siglo XXI, IMSS, which also approved of their use for these experiments. All experiments carried out with cells from human donors were performed following the Ethical Guidelines of the Instituto de Investigaciones Biomédicas, UNAM, Ciudad de México, México. PBMCs were isolated from buffy coats by gradient centrifugation with Lymphoprep. PBMCs were washed three times with PBS, pH 7.4, and were seeded (8–10 × 10^7^ PBMCs/plate) in 100 mm × 20-mm cell culture-treated polystyrene culture dishes (Corning 430167, New York, NY, USA), in RPMI-1640 medium supplemented with 10% (v/v) heat-inactivated autologous plasma-derived serum, 1 mM sodium pyruvate solution, 2 mM MEM non-essential amino acid solution, 0.1 mM l-glutamine, 100 U/mL penicillin, and 100 μg/mL streptomycin for 1 h at 37°C in a humidified atmosphere with 5% CO_2_, to allow monocytes to adhere to the plastic plate. Non-adherent cells were eliminated by washing, and adherent cells, enriched for monocytes (≥95% purity, as determined by flow cytometry by use of CD14 as a marker of the monocytic population), were cultured for 6 days for differentiation into macrophages, in RPMI-1640 medium supplemented with 10% (v/v) heat-inactivated FBS, 1 mM sodium pyruvate solution, 2 mM MEM non-essential amino acid solution, 0.1 mM l-glutamine, 100 U/mL penicillin, 100 μg/mL streptomycin, and recombinant human (rh) M-CSF at 5 ng/mL, at 37°C in a humidified atmosphere with 5% CO_2_. The resulting hMDMs were polarized by incubation with rhIFN-γ (30 ng/mL), or rhIL-4 (50 ng/mL), or rhIL-10 (20 ng/mL) for 48 h. The concentration of the cytokines was established in dose–response experiments. For experiments, polarized or non-polarized macrophages were harvested by gentle pipetting. Less than 1% cell death was observed in all conditions. Macrophages from each different donor were polarized in independent experiments. Non-polarized macrophages are referred to as M0 macrophages, and hMDMs polarized with IFN-γ, IL-4, or IL-10 are referred to as Mϕ-IFN-γ, Mϕ-IL-4, or Mϕ-IL-10, respectively.

### Expression of Surface Molecules by Flow Cytometry

Expression of surface markers on hMDMs was analyzed by flow cytometry (Attune Acoustic Focusing Flow Cytometer, Applied Biosystem, Foster City, CA, USA). Fluorochrome-labeled monoclonal antibodies specific for CD14, CD11b, CD11c, CD80, CD86, CD206, CD209, CD163, CD64, CD32, CD16, and CD13 were used. Equivalent concentrations of matched isotype controls were included. Before staining, Fc receptors were blocked with 10% autologous human serum. Cells were fixed in 1% paraformaldehyde and analyzed by flow cytometry. The surface expression levels of each marker were measured on polarized and non-polarized macrophages of each individual donor. The panel of surface molecules was selected based on the reports of human cells (22–31), as well as potential involvement of specific molecules in macrophage activation. Data were analyzed with Attune^®^ Cytometric Software version 1.2.5, compatible with both Blue/Violet and Blue/Red configurations. Values are expressed as the mean fluorescence intensity (MFI) of the marker of interest and as the ratio of the MFI of the marker over the MFI of the same marker on non-polarized cells from the same donor.

### RNA Isolation, DNase Treatment, and cDNA Synthesis

Polarized or non-polarized macrophages (3 × 10^6^) were harvested and lysed in TRIZOL (Invitrogen). Total RNA was extracted according to the manufacturer’s protocol. The precipitated RNA was dissolved in RNase-free water. The quality of the RNA was assessed by measuring the ratio of absorbance at 260 and 280 nm and by visualization of the integrity of the 28S and 18S bands in agarose gels. RNA samples were treated with DNase to remove contaminating DNA. Briefly, 10 μg total RNA was treated with TURBO DNase for 30 min at 37°C. Digestion was stopped by addition of DNase inactivation reagent, for 5 min at room temperature. The samples were centrifuged, and the supernatant containing RNA was recovered. For first-strand synthesis of cDNA from RNA molecules, 1 μg RNA was incubated with oligo-dT 12–18 primer for 5 min at 70°C, and dNTPs and M-MLV-RT were added. The mixture was incubated for 60 min at 37°C and for 15 min at 75°C.

### Gene Expression Analysis by Quantitative Real-time PCR

Quantitative real-time PCR was performed using gene-specific primers designed using Primer Express (Applied Biosystems). The primers are shown in Table 1. Quantitative RT-PCR (qRT-PCR) analyses were set up using 1.0 μL cDNA, 5 μL of SYBR^®^Green PCR Master Mix (Life Technologies), 0.25 μL of each forward and reverse primer (250 nM), 0.2 μL of uracil-*N*-glycosylase (Applied Biosystems), and 3.5 μL of injectable water, totalizing a final volume of 10 μL. Reactions were run in a 7500 Fast Real-Time PCR system (Applied Biosystems) under the following conditions: 50°C for 2 min, 95°C for 10 min, 40 cycles of 95°C for 15 s, and 60°C for 1 min. Melting curve analysis was carried out at the end of each PCR to confirm the specificity of PCR products.

**Table 1:** **Primers pairs used for determination of gene expression by qRT-PCR**.

Gene	Specific primers pair	bp	Exon
HPRT	Forward: TTATGGACAGGACTGAACGTCTTG	24	2–3
	Reverse: CCAGCAGGTCAGCAAAGAATT	21	
		Product: 114 bp	
FCGR1	Forward: GGGCAAGTGGACACCACAA	19	1–2
	Reverse: TGCAAGGTTACGGTTTCCTCTT	22	
		Product: 83 bp	
FCGR2A	Forward: GGCTTCTGCAGACAGTCAAGC	21	2–3
	Reverse: CCTGGAGCACGTTGATCCAC	20	
		Product: 80–77 bp	
FCGR2B	Forward: GCAGTTCCAAAAGAGAAGGTTTCT	24	8
	Reverse: TCGGTTATTTGGGACCATATTGT	23	
		Product: 97 bp	
FCGR3A	Forward: GGTGCAGCTAGAAGTCCATATCG	23	4–5
	Reverse: GAATAGGGTCTTCCTCCTTGAACA	24	
		Product: 77 bp	

Results were analyzed using the 7500 software (7500/7500 Fast Real-time PCR System) and were normalized using the endogenous gene *HPTR-1* and the ΔΔcycle threshold method. Data are expressed in terms of relative mRNA levels in polarized macrophages to mRNA levels in non-polarized cells (M0). Ten replicates per experimental condition were performed, and differences were assessed with one-way ANOVA test with Tukey *post hoc* test.

### Cytokine Secretion

To analyze the cytokine secretion profile of non-stimulated polarized macrophages, hMDMs were treated or not treated with rhIFN-γ, rhIL-4, or rhIL-10 for 48 h. The cells were collected and washed three times with PBS, pH 7.4; fresh media was added, and the cells were incubated for additional 24 h. The cell-free culture supernatants were collected and used to quantitatively measure IL-8, IL-1β, IL-6, IL-10, TNF-α, and IL-12p70 protein levels using the Cytometric Bead Array (CBA) Human Inflammatory Cytokines Kit. To determine the cytokine secretion by stimulation, polarized macrophages were stimulated for 24 h with LPS, a ligand for cell surface TLR4. The cell-free culture supernatants were used to quantitatively measure IL-8, IL-1β, IL-6, IL-10, TNF-α, and IL-12p70 protein levels by CBA. The assays were performed according to the manufacturer’s instructions and analyzed using flow cytometry. The amount of each cytokine in the supernatant was extrapolated using a standard curve based of the known amounts of the recombinant cytokine. The concentrations of the standards ranged from 20 to 5,000 pg/mL.

### Phagocytosis through FcγRI, FcγRII, or CD13 (Selective Phagocytosis)

Sheep red blood cells (SRBCs) were maintained in Alsever’s solution until used. Modified SRBCs were prepared as described previously (14). In brief, erythrocytes (at 1.2 × 10^9^/mL in PBS-BSA 0.1%) were stained with 10 mM CFSE. The stained SRBCs were incubated with 250 μg/mL Sulfo-NHS-biotin for 20 min at 4°C. After washing, they were coated with 35 μg/mL streptavidin for 20 min at 4°C. The biotin-streptavidin-coated erythrocytes were washed and incubated with biotinylated F(ab′)2 fragments of goat anti-mouse IgG for 30 min. SRBCs labeled with CFSE and coated with biotin, streptavidin, and fragments of biotinylated anti-IgG antibodies are henceforth designated EBS-Fab. For phagocytosis assays, 1 × 10^6^ hMDMs were incubated with 2 μg of Fab fragments of mAb452 (anti-human CD13), or 4 μg Fab fragments of mAb32.2 (anti-human FcγRI), or 4 μg Fab fragments of mAbIV.3 (anti-human FcγRII), or 4 μg IgG1 (isotype-matched control), or without treatment (control) for 30 min at 4°C, washed, and incubated with EBS-Fab at a ratio of 1 monocytic cell:20 EBS-Fab, at 37°C for 30 min. Equivalent samples were incubated at 4°C as negative controls of phagocytosis. Non-internalized erythrocytes were lysed by hypotonic shock. Phagocytosis was quantified by flow cytometry (Attune acoustic focusing flow cytometer; Applied Biosystems, Foster City, CA, USA), with addition of Trypan blue 0.02% in PBS 1× (pH 4.5), to quench extracellular fluorescence from attached but not internalized erythrocytes. Data are expressed as the percentage of CFSE-positive cells (i.e., cells that have ingested at least one erythrocyte) and as phagocytic index (PI), calculated using the following formula: PI = (% CFSE-positive cells) × (MFI of cells containing erythrocytes). Results were analyzed using Attune^®^ Cytometric Software version 1.2.5, compatible with both Blue/Violet and Blue/Red configurations.

### Phagocytosis of *Escherichia coli* and Zymosan Particles

Human monocyte-derived macrophages were treated or not treated with rhIFN-γ, rhIL-4, or rhIL-10 for 48 h. The hMDM suspension (1 × 10^6^ polarized or non-polarized cells) was incubated with 20 μL of fluorescein-conjugated heat-killed *E. coli* suspension or with 40 μg/mL of fluorescein-conjugated zymosan particles for 30 min at 37°C. Negative controls were prepared in identical conditions but incubated at 4°C. Phagocytosis was stopped by washing the suspension of hMDM and bacteria or zymosan particles with ice-cold PBS. Each sample was analyzed immediately after addition of Trypan Blue 0.02% in PBS 1× (pH 4.5), to quench extracellular fluorescence from attached but not internalized bacteria or particle. Data are expressed as the percentage of FITC-positive cells. Results were analyzed using Attune^®^ Cytometric Software version 1.2.5, compatible with both Blue/Violet and Blue/Red configurations.

### Quantification of ROS Production

Human monocyte-derived macrophages, non-polarized or treated with rhIFN-γ, rhIL-4, or rhIL-10, were collected and washed with HBSS. Cell suspensions (1 × 10^6^ hMDM) were incubated for 30 min at 4°C in HBSS with 2 μg of Fab fragments of mAb452 (anti-CD13), or 4 μg Fab fragments of mAb32.2 (anti-human FcγRI), or 4 μg Fab fragments of mAbIV.3 (anti-human FcγRII), or 4 μg murine IgG1 (isotype-matched control), or in HBSS alone (control No Fab). After a brief centrifugation, the supernatant was discarded, and the cells were loaded with a cell-permeable ROS-sensitive fluorescent dye, by incubating with 20 mM carboxy-H2DFFDA, for 30 min at 37°C in HBSS. After washing, the receptors FcγRI, FcγRII, and CD13 were crosslinked for 30 min at 37°C by incubation with EBS-Fab at a ratio of 1 cell:20 EBS-Fab. To maximize contact, the cells mixed with the EBS-Fab were pelleted by a brief centrifugation. The cells were resuspended and transferred to wells of black 96-well Immuno Plates (Thermo Scientific, Waltham, MA, USA). Fluorescence from oxidized carboxy-H2DFFDA was determined immediately (initial reading) and every 15 min thereafter, in a Cytation 3 Cell Imaging Multi-Mode Reader (BioTek, Winooski, VT, USA) for 60 min. For quantification of ROS production in M2 macrophages, cells were preincubated on ice with two different Fab fragments [anti-FcγRI (32.2) + anti-FcγRII (IV.3), or anti-FcγRI (32.2) + anti-CD13 (452)], or with three Fab fragments (32.2 + IV.3 + 452), or no treatment (No Fab). Subsequently, cells were washed and loaded with carboxy-H2DFFDA. Cells were mounted in Black 96-well plates and read immediately and every 30 min thereafter, for 150 min.

### Statistical Analysis

**Figure 1:**
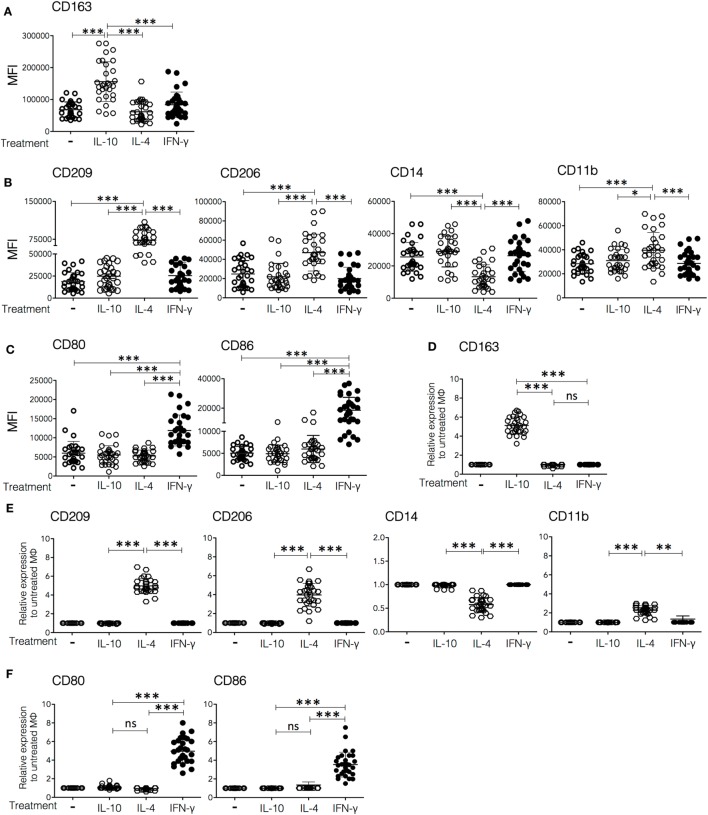
**Cell surface markers expressed on *in vitro* polarized macrophages**. Monocytes from peripheral blood of healthy donors were cultured for 6 days in medium containing macrophage colony-stimulating factor to differentiate into M0. The resulting human monocyte-derived macrophages were polarized by incubation with IFN-γ (30 ng/mL), IL-4 (50 ng/mL), or IL-10 (20 ng/mL) for 48 h and subsequently analyzed by flow cytometry for the expression of surface markers. **(A–C)** Mean fluorescence intensity (MFI) of each marker in non-polarized and polarized cells from 30 individual donors. **(D–F)** Fold increase in MFI relative to control (non-polarized macrophages) of cells from 30 individual donors polarized with IFN-γ, IL-4, or IL-10. Plots are grouped in lines to show **(A,D)** markers upregulated in Mϕ-IL-10 (upper plot), **(B,E)** markers upregulated or downregulated in Mϕ-IL-4 (middle-line plots), and **(C,F)** markers upregulated in Mϕ-IFN-γ (lower line plots). **(A–C)** One-way ANOVA was used to compare among M0, Mϕ-IFN-γ, Mϕ-IL-4, and Mϕ-IL-10 followed by Tukey’s *post hoc* test for comparisons between treatment groups. For analysis of the expression of surface markers using normalization of data **(D–F)**, ANOVA was used to compare among Mϕ-IFN-γ, Mϕ-IL-4, and Mϕ-IL-10, followed by Tukey’s *post hoc* test for comparisons between treatment groups (**p < 0.01 and ***p < 0.001).

**Figure 2:**
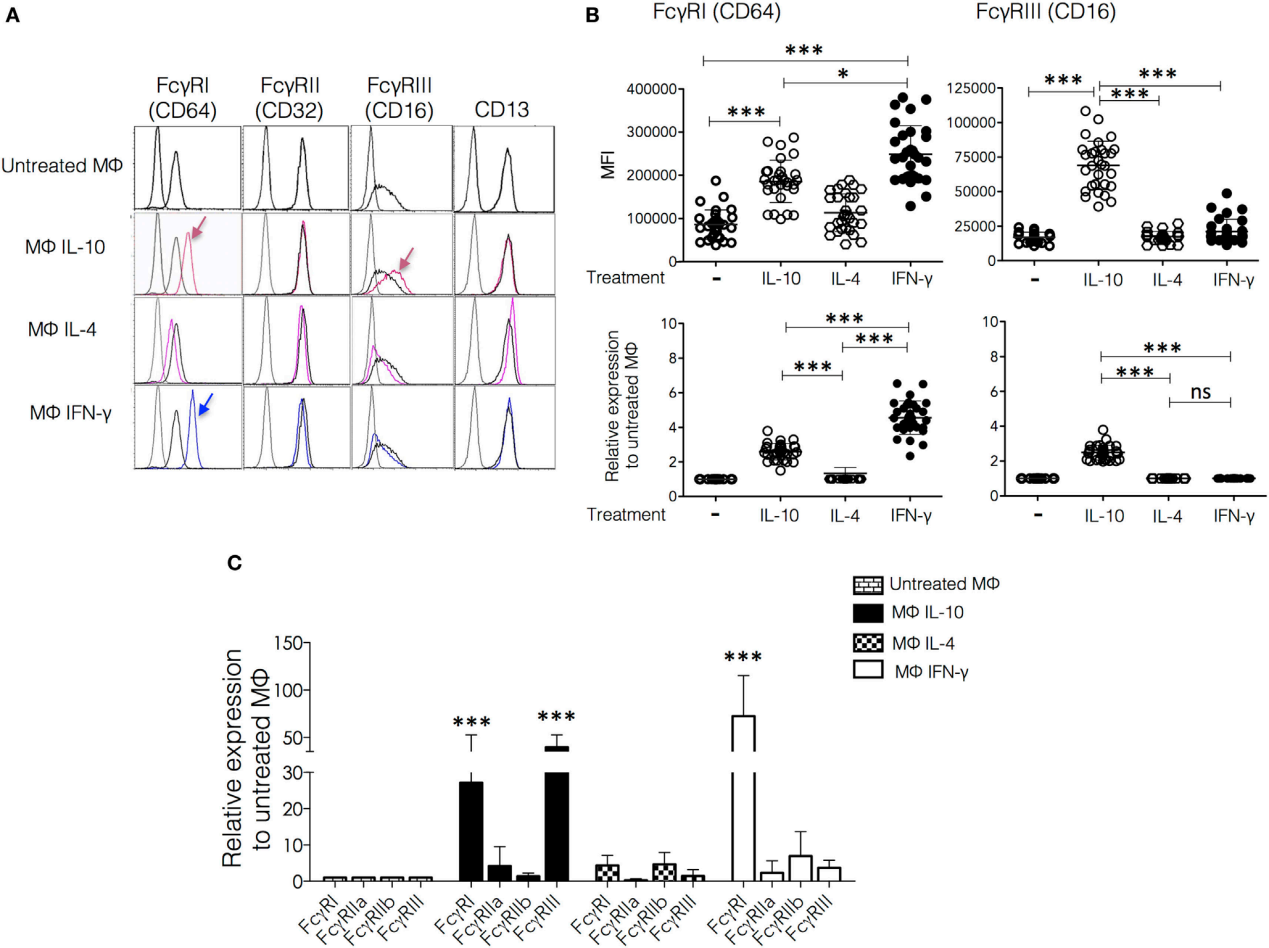
**Effect of polarization on membrane expression of FcγRs and CD13**. Monocytes from healthy donors were cultured for 6 days in a medium supplemented with macrophage colony-stimulating factor (M-CSF) to differentiate into M0. The resulting human monocyte-derived macrophages were polarized by incubation with IFN-γ (30 ng/mL), IL-4 (50 ng/mL), or IL-10 (20 ng/mL) for 48 h and subsequently analyzed by flow cytometry for the expression of FcγRI, FcγRII, FcγRIII, and CD13. **(A)** Representative histograms of cells from a single donor. The arrows indicate significant changes in receptor expression induced by polarization. Colored histograms are from cytokine-treated cells. **(B)** Upper plots show the mean fluorescence intensity (MFI) of FcγRI and FcγRIII in non-polarized and polarized cells from 30 individual donors, and lower plots show the same data plotted as average fold increase in MFI relative to control (non-polarized macrophages or M0). **(C)** After polarization, cells were lysed, and RNA was isolated for quantification of mRNA for *Fc*γ*RI, Fc*γ*RIIa, Fc*γ*RIIb*, and *Fc*γ*RIII* by real-time PCR. Average fold increase of mRNA relative to non-polarized macrophages in cells from 10 different donors analyzed in triplicate. Results are expressed as mean + SD of independent experiments. Statistical significance was calculated using one-way ANOVA with Tukey *post hoc* test. For analysis of the expression of FcγRs using normalization of data [**(B)**, lower plots] ANOVA was used to compare Mϕ-IFN-γ, Mϕ-IL-4, and Mϕ-IL-10, followed by Tukey’s *post hoc* test for comparisons between treatment groups (*p < 0.05 and ***p < 0.001).

Statistical analysis was performed using GraphPad-Prism software (GraphPad, La Jolla, CA, USA). Data are expressed as the mean ± SD. One-way ANOVA was used to compare among M0, Mϕ-IFN-γ, Mϕ-IL-4, and Mϕ-IL-10, followed by Tukey’s *post hoc* test for comparisons between groups. Statistical significance was considered with *p* < 0.05. For analysis of the expression of surface markers using normalization of data (Figures 1D–F; Figure 2B, lower plots), ANOVA was used to compare among Mϕ-IFN-γ, Mϕ-IL-4, and Mϕ-IL-10, followed by Tukey’s *post hoc* test for comparisons between treatment groups.

## Results

### Distinct Cell Surface Markers Expression by Polarized Macrophages

To characterize phenotypically the macrophage populations polarized *in vitro*, we determined the expression of a panel of surface molecules by flow cytometry after 2 days of *in vitro* polarization with rhIFN-γ, rhIL-4, or rhIL-10, using non-polarized cells (M0) as a control. The cell surface molecules analyzed were CD163, CD209, CD206, CD14, CD11b, CD11c, CD80, and CD86; these markers were selected based on literature reports, and the involvement of these molecules in macrophage activation (22–33). Figures 1A–C show the MFIs for each marker observed in cells from 30 different donors, whereas Figures 1D–F show the same data expressed as the ratio of the MFI of the marker of interest on cytokine-polarized cells over the MFI of the same marker on non-polarized cells from the same donor, to show more clearly the effect of each treatment on membrane expression of the markers. Macrophages treated with IL-10 (Mϕ-IL-10) showed a specific upregulation of the expression of the scavenger receptor CD163 (mean 5.2-fold increase) (*p* < 0.001), as compared to non-polarized and IL-4 or IFN-γ polarized macrophages (Figures 1A,D; Figure S1 in Supplementary Material). IL-4 induced the expression of CD209 [dendritic cell-specific ICAM-3-grabbing non-integrin (DC-SIGN)], a C-type lectin (mean 4.9-fold increase) (*p* < 0.001) and an increase in the expression of CD206 (mannose receptor C-type 1) (mean 3.9-fold increase) (*p* < 0.001). CD209 is expressed neither in M0 macrophages nor in Mϕ-IFN-γ or Mϕ-IL-10 and therefore is a useful marker for Mϕ-IL-4 (34). Also, expression of CD11b is increased (mean 2.3-fold increase) (*p* < 0.001), and expression of CD14 is significantly decreased (to an average of half the value of M0 cells) (*p* < 0.001) in Mϕ-IL-4, compared with M0, Mϕ-IFN-γ, or Mϕ-IL-10 (Figures 1B,E; Figure S1 in Supplementary Material). Finally, Mϕ-IFN-γ displayed a robust and specific upregulation of the costimulatory molecules CD80 (mean 4.9-fold) (*p* < 0.001) and CD86 (mean 3.6-fold) (*p* < 0.001) compared to M0, Mϕ-IL-4, and Mϕ-IL-10 (Figures 1C,F; Figure S1 in Supplementary Material). We did not find significant differences in CD11c expression among the different subpopulations of macrophages (Figure S1 in Supplementary Material). In summary, human Mϕ-IL-10 are characterized by high expression of CD163; Mϕ-IL-4 specifically upregulates CD209, CD206, and CD11b and downregulate CD14; and Mϕ-IFN-γ specifically upregulates the expression of the costimulatory molecules CD80 and CD86.

Once validated the surface markers for Mϕ-IL-10, Mϕ-IL-4, and Mϕ-IFN-γ polarized *in vitro*, we investigated the effect of polarization on membrane expression of FcγRs and CD13. Figure 2A shows representative histograms, and Figure 2B shows plots of the MFI values, both as MFI and as relative expression normalized to the expression on non-polarized cells from the same donor. Mϕ-IL-10 showed a specific upregulation of FcγRIII (CD16) (mean 2.4-fold increase) compared to all other macrophage populations (*p* < 0.001). Mϕ-IL-10 also showed an increased expression of the high-affinity FcγRI (CD64) compared to M0 or Mϕ-IL-4 (mean 2.5-fold increase) (*p* < 0.001) (Figures 2A,B). CD64 was also significantly increased in Mϕ-IFN-γ (CD64^high^) compared to all other macrophages populations (mean 4.5-fold increase with respect to M0) (Figures 2A,B). Although both IL-10 and IFN-γ induced an increased expression of CD64 in comparison to non-polarized macrophages (*p* < 0.001), the expression induced by IFN-γ is significantly higher than the expression induced by IL-10 (*p* < 0.05) (Figures 2A,B). We did not find significant differences in FcγRII (CD32) or CD13 expression among different populations of macrophages (Figure 2A). These results suggest that the expression levels of FcγRs (but not of CD13) are differently modulated by the macrophage activation phenotype.

The effects of the polarizing cytokines on the levels of mRNA coding for FcγRs were evaluated by quantitative RT-PCR. Compared to the non-polarized macrophages (M0), Mϕ-IFN-γ have significantly higher FcγRI (CD64) mRNA levels (FcγRI, mean 72.5-fold increase, *p* < 0.001) (Figure 2C). In Mϕ-IL-10, mRNA levels of FcγRI and FcγRIII (CD16) were significantly increased (FcγRI, mean 27.1-fold increase, *p* < 0.01; FcγRIII, mean 39.7-fold increase, *p* < 0.001). The elevated mRNA level of FcγRI observed in macrophages treated with IFN-γ correlates with the increased membrane expression (Figure 2B). Likewise, the increased mRNA levels of FcγRI and FcγRIII in Mϕ-IL-10 (Figure 2C) agree with the increases in membrane expression of these receptors (Figure 2B). Thus, IFN-γ and IL-10 modulate the expression of FcγRs at the mRNA level.

All populations of macrophages express substantial levels of FcγRII on their membrane (Figure 2A). Although the histograms show no difference among the different populations, the RT-PCR analysis indicates differences in the ratio of FcγRIIa/FcγRIIb among the polarized macrophages (Figure 2C). This ratio is higher in Mϕ-IL-10 and lower in Mϕ-IL-4 and Mϕ-IFN-γ. Because the FcγRIIa isoform contains in its intracellular portion an activatory ITAM, while FcγRIIb contains an inhibitory ITIM, these differences are likely to be important in the triggering of effector functions through stimulus that engage FcγRII.

### Cytokine Secretion by Polarized Macrophages

**Figure 3:**
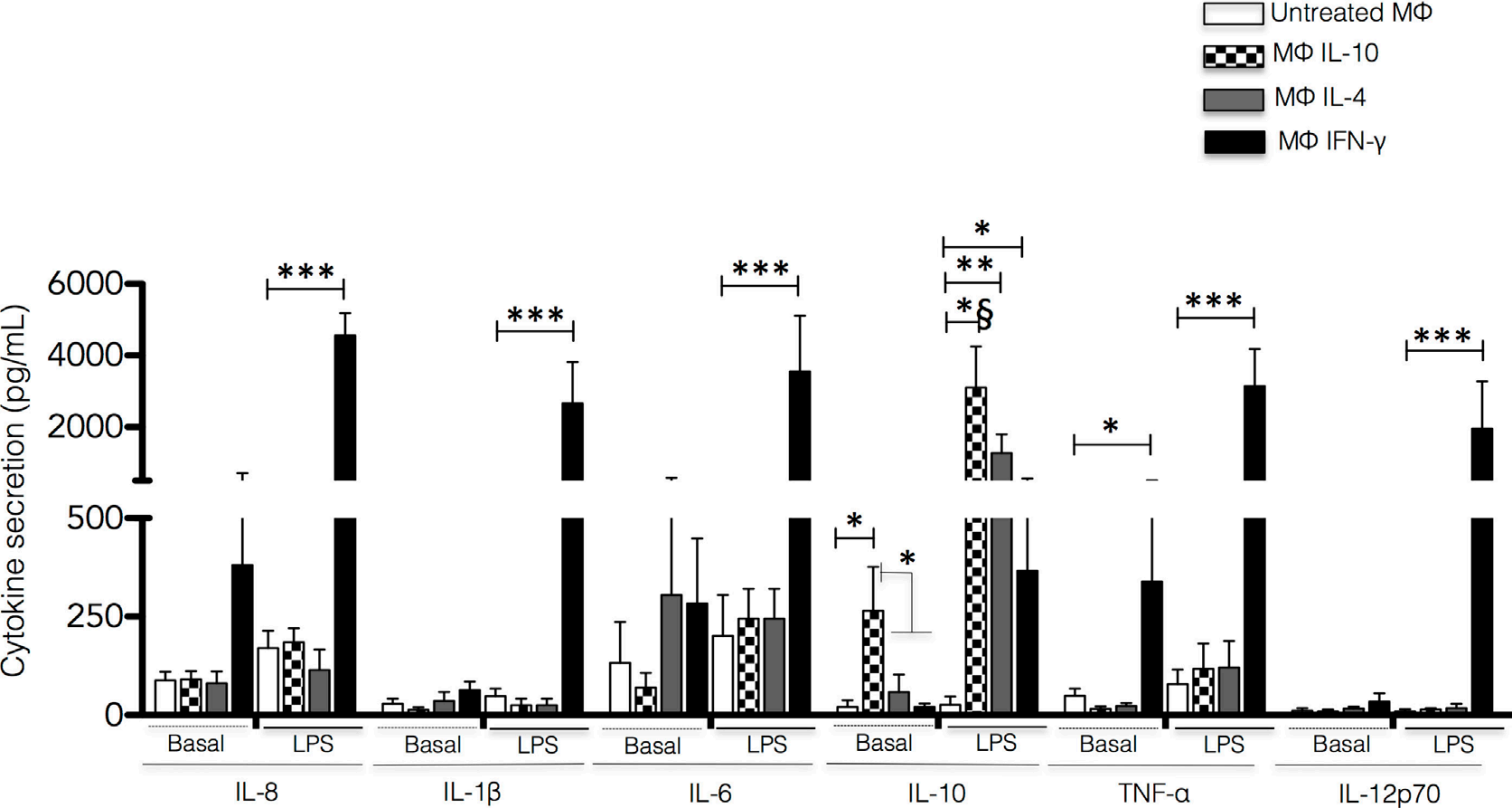
**Cytokine secretion by non-polarized and polarized macrophages**. Human monocyte-derived macrophages were polarized by incubation with IFN-γ, IL-4, or IL-10 for 48 h. The polarizing stimulus was completely removed by washing, and the cells were incubated for additional 24 h in a fresh medium with or without LPS. The cell culture supernatants were recovered, and the concentrations of IL-8, IL-1β, IL-6, IL-10, TNF-α and IL-12p70 were measured by Cytometric Bead Array. Results are expressed as mean + SD of independent experiments performed in triplicate with cells from 15 different donors. Statistical significance was calculated using one-way ANOVA with Tukey *post hoc* test (*p < 0.05, **p < 0.01, *** and *^§^*p* < 0.001).

To analyze the cytokine secretion profile of the different populations of polarized macrophages, the polarizing cytokines were completely removed by washing after 48 h. Fresh media with or without LPS was added, and the cells were incubated for additional 24 h. Cell-free culture supernatants were used for determination of the production of IL-8, IL-6, IL-1β, IL-10, TNF-α, and IL-12p70. Unstimulated Mϕ-IL-10 secreted a low, yet significant amount of IL-10 (*p* < 0.05), whereas unstimulated Mϕ-IFN-γ produced significant levels of TNF-α compared to the other macrophage populations analyzed (*p* < 0.05) (Figure 3). In the absence of stimulation, Mϕ-IL-4 macrophages did not produce significant levels of any cytokine examined (Figure 3).

After stimulation with LPS for 24 h, macrophage subsets showed distinct profiles of cytokine production. In Mϕ-IFN-γ, LPS stimulation induced significant secretion of IL-8, IL-6, TNF-α, IL-1β, and IL-12p70 compared to non-polarized macrophages and macrophages treated with IL-4 or IL-10 (all *p* < 0.001) (Figure 3). In contrast, upon LPS stimulation, Mϕ-IL-10 and Mϕ-IL-4 secreted significantly higher amounts of IL-10 (*p* < 0.001 and *p* < 0.01, respectively) compared to M0 macrophages. Mϕ-IFN-γ macrophages also produced significant levels of IL-10 upon stimulation with LPS (*p* < 0.05) although to a lesser degree than Mϕ-IL-10 and Mϕ-IL-4 macrophages (Figure 3). Thus, macrophages activated to different phenotypes exhibited a specific cytokine profile in both resting state and response to LPS stimulation.

### M0 and Mϕ-IL-10 Macrophages Show Higher FcγR-Mediated Phagocytosis

To determine whether differences in the expression of FcγRs observed among the different subpopulations of *in vitro* polarized macrophages (Figure 2) are reflected at the functional level, we examined phagocytosis mediated by FcγRs in non-polarized and polarized macrophages.

We used an experimental system to direct sheep erythrocytes (as the phagocytic prey) to individual receptors on the cell, as reported previously (14). Erythrocytes loaded with CFSE and labeled with biotin and streptavidin were coated with biotin-F(ab′)2 fragments of goat anti-mouse IgG (EBS-Fab). These EBS-Fabs specifically interact with the molecules on the cell surface tagged with bound Fab fragments of specific murine mAb. As the specificity of the system is based on antibodies and macrophages express FcγRs, we used Fab fragments to exclude any possible contribution of Fc fragments binding to FcγRs to the phagocytosis. Cells were preincubated on ice with Fab fragments of anti-FcγRI mAb32.2 (Fab32.2) or Fab fragments of anti-FcγRII mAb IV.3 (Fab IV.3) or no treatment (basal phagocytosis, designated as No Fab). Cells were washed, mixed with EBS-Fab, and incubated at 37 or 4°C for 30 min. After incubation, non-internalized erythrocytes were lysed by hypotonic shock. The percentage of cells with internalized erythrocytes (CFSE-positive cells) and the MFI of the CFSE-positive cells was determined by flow cytometry after quenching extracellular fluorescence with Trypan blue.

**Figure 4:**
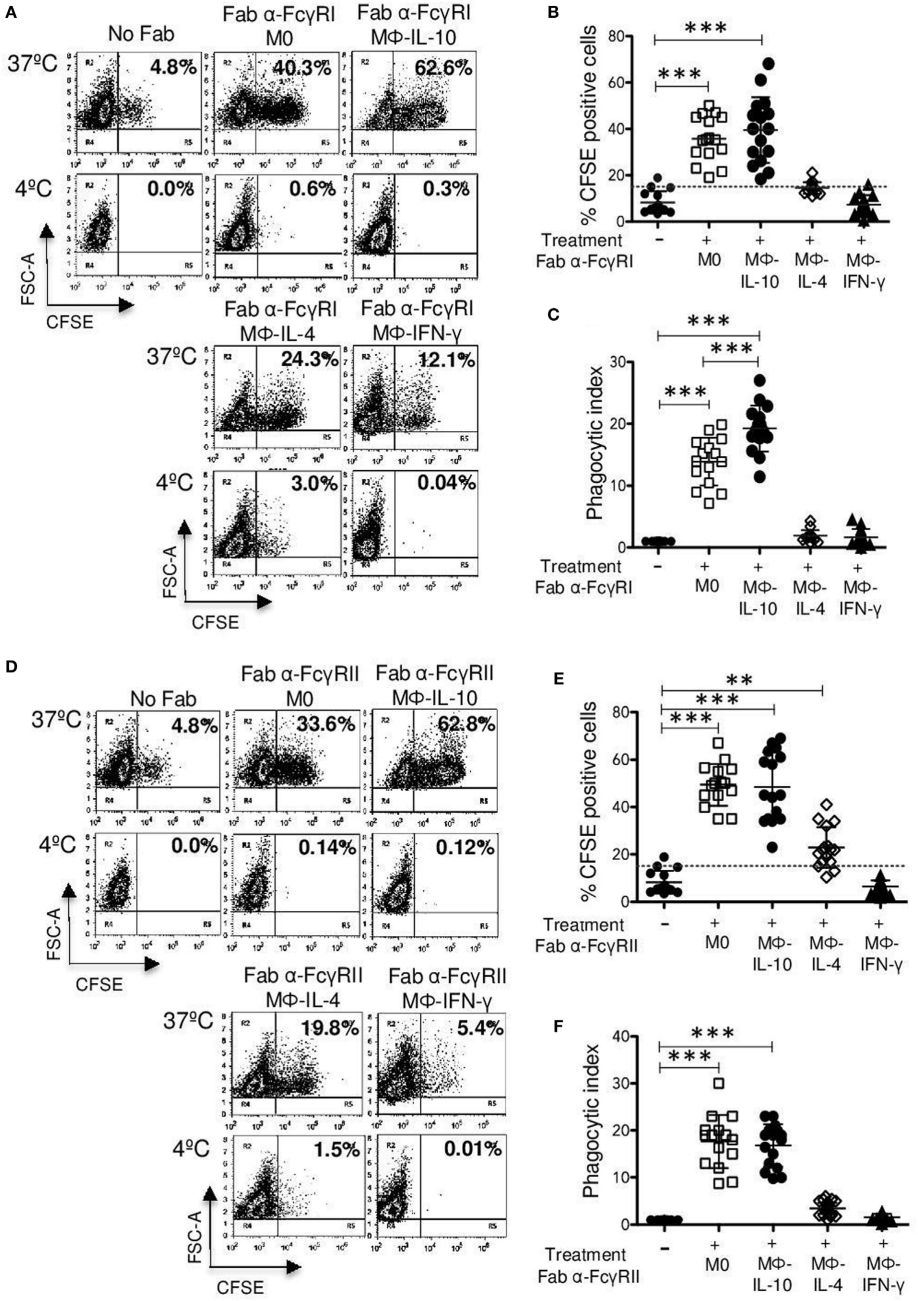
**Continued FcγRI- and FcγRII-mediated phagocytosis in non-polarized and polarized human macrophages**. Polarized and non-polarized macrophages were incubated with 4 μg Fab fragments of mAb32.2 (Fab 32.2; anti-FcγRI) or Fab fragments of mAbIV.3 (Fab IV.3; anti-FcγRII) or without antibody (No Fab) for 30 min at 4°C. After washing, macrophages were incubated for 30 min at 37 or 4°C with carboxyfluorescein succinimidyl ester (CFSE)-labeled F(ab′)2 goat anti-mouse-opsonized erythrocytes (EBS-Fab). Non-internalized erythrocytes were lysed, and samples were analyzed by flow cytometry to determine the percentage of CFSE-positive cells. **(A)** Representative dot plots of a single experiment, showing FcγRI-mediated phagocytosis. **(B)** Average of CFSE-positive cells (M0 and polarized macrophages) treated with Fab fragment 32.2 (anti-FcγRI) and incubated with EBS-Fab (*n* = 15). **(C)** Phagocytic index (PI) (geometric mean of fluorescence intensity multiplied by the percentage of positive cells) of FcγRI-mediated phagocytosis in M0 and polarized macrophages. Data were normalized considering the value obtained in the absence of FcγRI crosslinking (No Fab) as 1. **(D)** Representative dot plot of a single experiment, showing FcγRII-mediated phagocytosis. **(E)** Average of CFSE-positive cells (M0 and polarized macrophages) treated with Fab fragment IV.3 (anti-FcγRII) and incubated with EBS-Fab (*n* = 15). **(F)** PI of FcγRII-mediated phagocytosis in M0 and polarized macrophages. Data were normalized considering the value obtained in the absence of FcγRII crosslinking as 1. Results are expressed as mean + SD of independent experiments. Statistical significance was calculated using one-way ANOVA with Tukey *post hoc* test (***p < 0.001).

**Figure 5:**
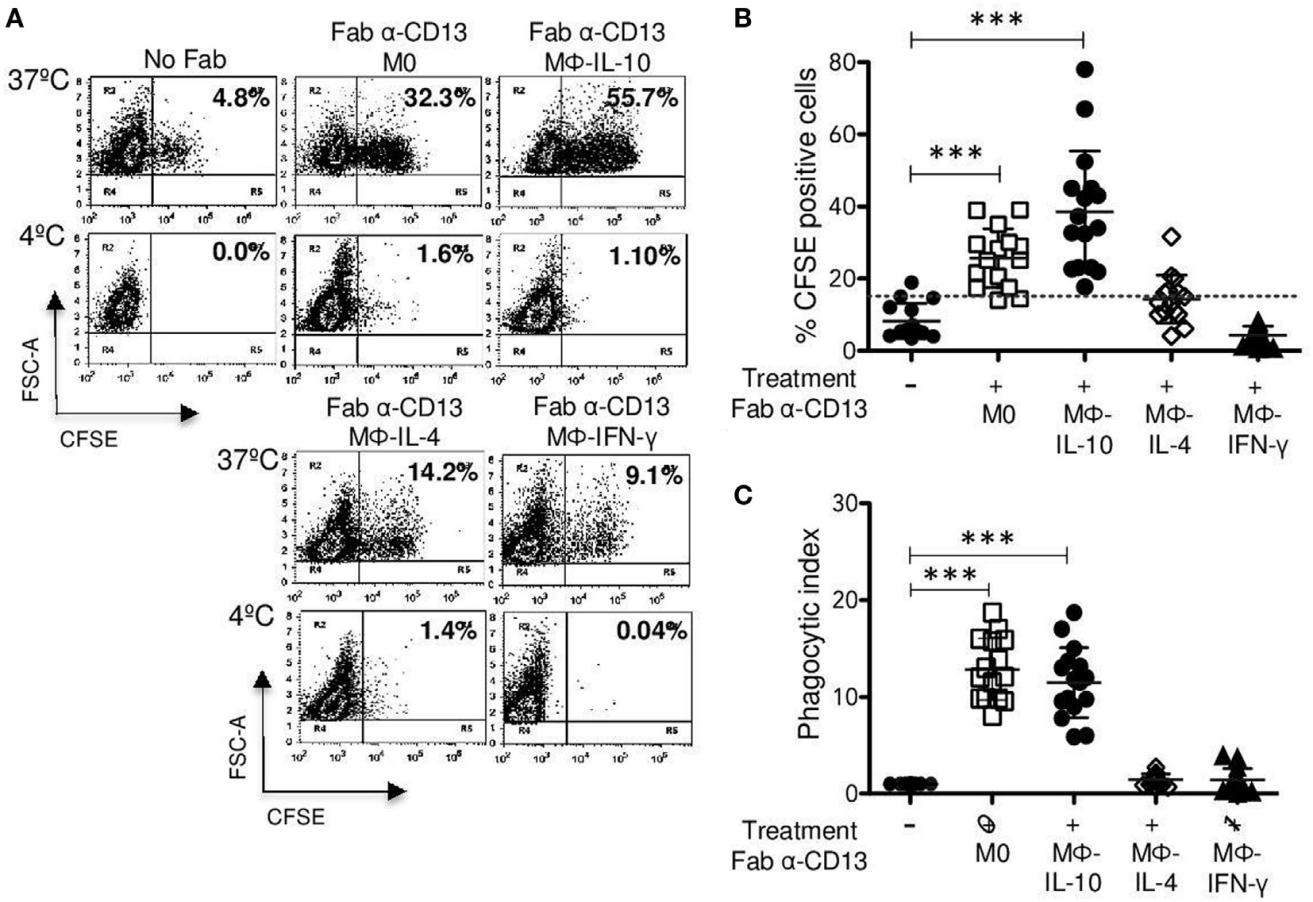
**CD13-mediated phagocytosis in non-polarized and polarized macrophages**. Non-polarized and polarized macrophages were incubated with 2 μg Fab fragments of mAb452 (anti-CD13) or without antibody (No Fab) for 30 min at 4°C. After washing, macrophages were incubated for 30 min at 37 or 4°C with carboxyfluorescein succinimidyl ester (CFSE)-labeled F(ab′)2 goat anti-mouse-opsonized erythrocytes (EBS-Fab). Non-internalized erythrocytes were lysed, and samples were analyzed by flow cytometry to determine the percentage of CFSE-positive cells. **(A)** Representative dot plots of a single experiment, showing CD13-mediated phagocytosis. **(B)** Average of CFSE-positive cells (M0 and polarized macrophages) treated with Fab of mAb 452 (anti-CD13) and incubated with EBS-Fab (*n* = 15). **(C)** Phagocytic index (geometric mean of fluorescence intensity multiplied by the percentage of positive cells) of CD13-mediated phagocytosis in M0 and polarized macrophages. Data were normalized considering the value obtained in the absence of CD13 crosslinking (No Fab) as 1. Results are expressed as mean + SD of independent experiments. Statistical significance was calculated using one-way ANOVA with Tukey *post hoc* test (***p < 0.001).

Binding of EBS-Fab to the cells through FcγRI induced its efficient internalization by Mϕ-IL-10 (mean 39.5%) (*p* < 0.001) and by non-polarized macrophages (M0) (mean 35.8%) (*p* < 0.001) compared with EBS-Fab internalization by cells not treated with Fabs (basal phagocytosis) (mean 8.26%; Figures 4A,B). Mϕ-IL-4 exhibited a poor internalization of EBS-Fab through FcγRI (mean 14.5%), which was not significantly different from basal phagocytosis. Mϕ-IFN-γ showed the lowest uptake of EBS-Fab (mean 7.2%), which was similar to phagocytosis of EBS-Fab by M0 cells with no Fab (basal phagocytosis) (Figure 4B). As expected, no significant internalization was observed when identical samples were kept at 4°C (Figure 4A, lower dot plots). To compare the PIs (PI = % CFSE-positive cells multiplied by mean fluorescence intensity), we first normalized the PI values of each sample to the basal phagocytosis of EBS-Fabs (cells not treated with Fabs) of cells from the same donor (which was given a value of 1). This was done to correct from differences in CFSE labeling of erythrocytes and in basal phagocytosis by cells from each different donor. Comparison of PI of phagocytosis mediated by FcγRI showed similar results to those obtained from comparing the percentage of CFSE-positive cells (Figure 4C). However, unlike the results of percentages, the mean PI of internalization through FcγRI by Mϕ-IL-10 (19.2-fold increase) (*p* < 0.001) was significantly higher than the mean PI of M0 (non-polarized) macrophages (13.8-fold increase) (Figure 5C). Thus, Mϕ-IL10 showed the highest phagocytosis mediated through FcγRI compared to all other macrophage populations, followed by non-polarized macrophages that were significantly more phagocytic than Mϕ-IL-4 and Mϕ-IFN-γ.

The binding of EBS-Fab to the cells through FcγRII induced its efficient internalization by non-polarized macrophages (M0) (mean 49.4%) (*p* < 0.001), Mϕ-IL-10 (mean 48.4%) (*p* < 0.001), and Mϕ-IL-4 (mean 22.8%) (*p* < 0.01) compared with no treatment (basal phagocytosis) (mean 8.26%; Figures 4D,E). Mϕ-IFN-γ did not internalize EBS-Fabs through FcγRII (mean 7.2%). No significant internalization by polarized or non-polarized macrophages was observed when identical samples were kept at 4°C (Figure 4D, lower plots). When we compared the PI, we observed similar results to those from comparing the percentages of CFSE-positive cells (Figure 4F), except that the mean PI of internalization through FcγRII by Mϕ-IL-4 (mean 3.47%) was not statistically different from basal phagocytosis. Likewise, Mϕ-IFN-γ (mean 1.6%) exhibited a PI similar to the basal phagocytosis (mean 1.0%) (Figure 4F). There were no significant differences in PIs between Mϕ-IL-10 (17.6-fold increase) and non-polarized macrophages (16.8-fold increase) for FcγRII-mediated phagocytosis. Thus, similar to the phagocytosis through FcγRI, both M0 and Mϕ-IL-10 showed significantly higher phagocytosis through FcγRII than Mϕ-IL-4 and Mϕ-IFN-γ. The low phagocytosis through FcγRII of Mϕ-IL-4 and Mϕ-IFN-γ is consistent with the low ratio of FcγRIIa/FcγRIIb in this population (Figure 2C).

We also evaluated phagocytosis of IgG-opsonized SRBC (involving the participation of all FcγRs expressed by the cell) by M0, Mϕ-IFN-γ, Mϕ-IL-4, and Mϕ-IL-10. All populations of macrophages internalized both non-opsonized and IgG-opsonized SRBC, but to different extents. M0 macrophages showed percentages of phagocytosis of non-opsonized SRBCs of 12.1% and of 52.9% for phagocytosis of IgG-opsonized SRBCs (Figure S2 in Supplementary Material). As reported previously (35), treatment of hMDM with IFN-γ resulted in a significantly reduced FcγRs-mediated phagocytosis (mean 14.8%) (*p* < 0.001) compared with M0 macrophages and also a lower phagocytosis of non-opsonized SRBC (mean 8.1%) (Figure S2 in Supplementary Material). In contrast, Mϕ-IL-10 showed a significantly higher phagocytosis of IgG–SRBC (mean 74.6%) (*p* < 0.001) and of non-opsonized SRBC (mean 15.4%). Mϕ-IL-4 showed an intermediate phagocytic capacity between that of Mϕ-IL-10 and that of Mϕ-IFN-γ, with percentages of 9.4 and 32.6% for phagocytosis of non-opsonized and IgG-opsonized SRBCs, respectively (Figure S2 in Supplementary Material). Similar to the results of phagocytosis through FcγRI and through FcγRII, both M0 and Mϕ-IL-10 show high phagocytic capacity for IgG-opsonized preys, with Mϕ-IL-10 exhibiting the highest phagocytic capacity compared to all other populations.

### M0 and Mϕ-IL-10 Macrophages Show Higher CD13-Mediated Phagocytosis

Even though we did not find differences in CD13 expression between different populations of macrophages (Figure 2A), we evaluated whether there were differences in CD13-mediated phagocytosis of EBS–Fab. The percentages of CFSE-positive cells of Mϕ-IL-10 (mean 38.5%) and non-polarized macrophages (M0) (mean 25.6%) were statistically significant (*p* < 0.001) compared with basal phagocytosis of EBS-Fab (mean 8.26%) (Figures 5A,B), while CD13-mediated phagocytosis by Mϕ-IL-4 (mean 14.3%) or Mϕ-IFN-γ (mean 4.3%) was not significantly different from non-specific phagocytosis of EBS-Fab (Figures 5A,B). No significant internalization was observed when identical samples were kept at 4°C (Figure 5A, lower dot plots). With respect to the PI of specific phagocytosis through CD13, we observed a significant increase in PI for Mϕ-IL-10 (12.8-fold increase) (*p* < 0.001) and non-polarized macrophages (11.4-fold increase) (*p* < 0.001) compared to basal phagocytosis (Figure 5B), while PIs for Mϕ-IL-4 (mean 1.4%) and Mϕ-IFN-γ (mean 1.4%) (Figure 5B) were no different to basal phagocytosis. No significant differences in PIs of CD13-mediated phagocytosis were observed between Mϕ-IL-10 and M0 (non-polarized) macrophages.

### Macrophage Activation Phenotypes Distinctively Affect Phagocytosis of Bacteria and Zymosan Particles

**Figure 6:**
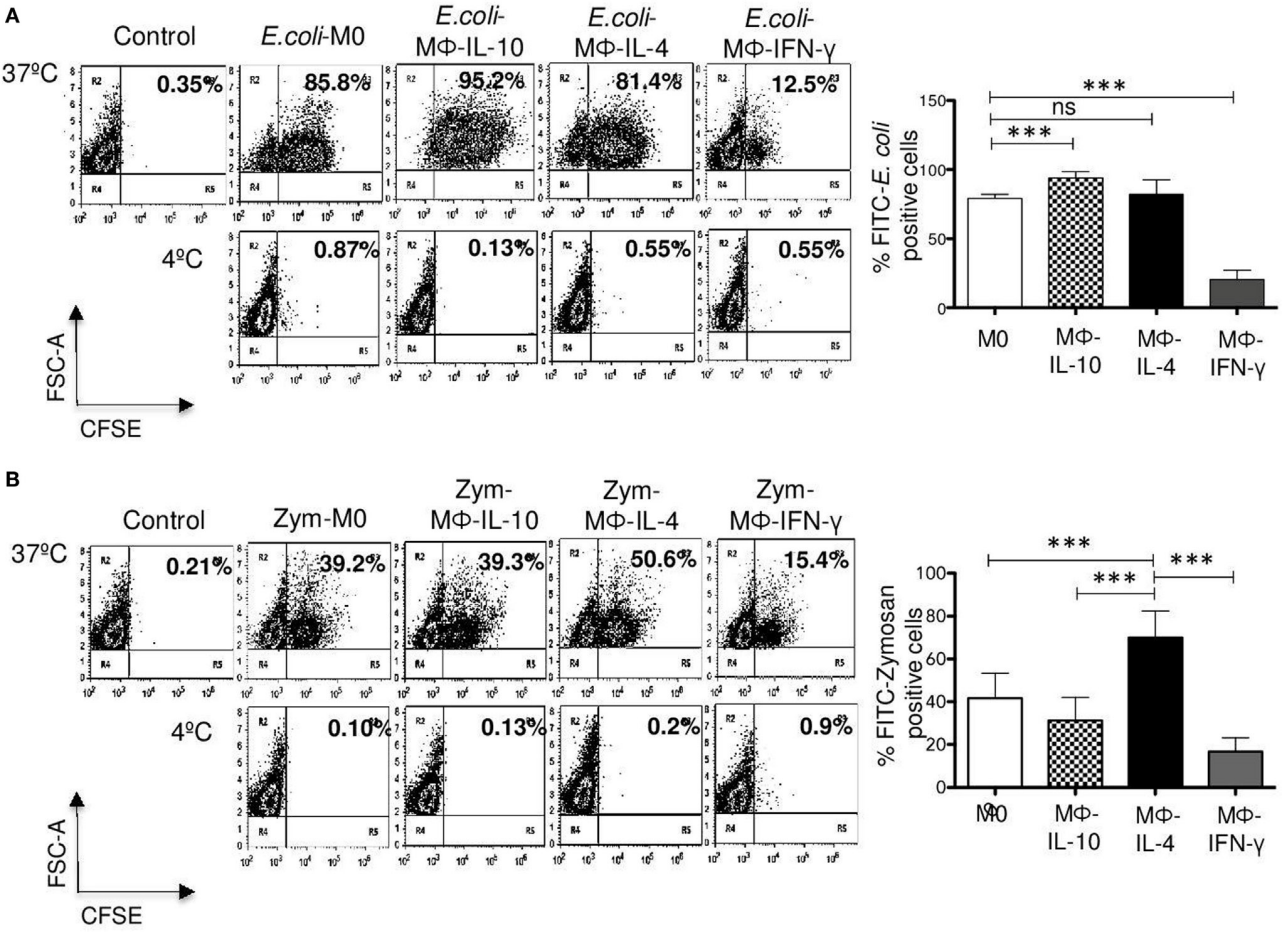
**Phagocytosis of *Escherichia coli* and zymosan in non-polarized and polarized macrophages**. M0 macrophages were polarized with IFN-γ, IL-4, or IL-10 for 48 h. After washing, macrophages were incubated for 30 min at 37 or 4°C with FITC-labeled *E. coli* or FITC-labeled zymosan particles. Non-internalized *E. coli* or zymosan were removed by washing, and samples were analyzed by flow cytometry to determine the percentage of FITC-positive cells in the presence of Trypan blue 0.02% in PBS, to quench extracellular fluorescence from attached but not internalized particles. **(A)** Representative dot plots of a single experiment, showing *E. coli* phagocytosis (left). Bar graph (right) show the average of FITC-*E. coli*-positive cells (*n* = 15). **(B)** Representative dot plot of a single experiment, showing phagocytosis of zymosan particles (left). Bar graph show the average of FITC-zymosan positive cells (*n* = 15) (right). Results are expressed as mean ± SD of independent experiments. Statistical significance was calculated using one-way ANOVA with Tukey *post hoc* test. ns, not significant; ***p < 0.001.

To determine whether the differences found in specific phagocytosis through FcγRI, FcγRII, and CD13 are also evident in phagocytosis through other receptors, we analyzed the effect of polarization on phagocytosis of FITC-labeled *E. coli* and FITC-labeled zymosan, using flow cytometry. M0, Mϕ-IL-10, and Mϕ-IL-4 efficiently internalized FITC-*E. coli*; Mϕ-IL-10 showed the highest percentage of phagocytosis (mean 93.9%) (*p* < 0.001) (Figure 6A), while Mϕ-IL-4 (mean 81.3%) showed no significant difference with phagocytosis by M0 macrophages (mean 79.1%) (Figure 6A). However, the percentage or phagocytic cells in Mϕ-IFN-γ (mean 20%) (*p* < 0.001) was significantly lower compared to non-polarized macrophages (M0) (mean 79.1%) (Figure 6A). No significant internalization was observed when identical samples were kept at 4°C (Figure 6A, lower dot plots). The reduced phagocytosis shown by Mϕ-IFN-γ cannot be attributed to a reduced binding of bacteria, since the binding of bacteria to Mϕ-IFN-γ (measured at 4°C) was higher compared with non-polarized macrophages and other populations of polarized macrophages (data not shown).

With respect to internalization of FITC-labeled zymosan particles, Mϕ-IL-4 exhibited the highest level of phagocytic cells (mean 69.9%) (*p* < 0.001) (Figure 6B). Significant phagocytic activity of zymosan was observed also for non-polarized macrophages (M0) (mean 41.6%) and Mϕ-IL-10 (mean 31.3%) (Figure 6B). Mϕ-IFN-γ macrophages showed reduced uptake of zymosan particles compared with M0 and Mϕ-IL-4 macrophages (Figure 6B) (*p* < 0.001). No significant internalization was observed when identical samples were kept at 4°C (Figure 6B, lower dot plots).

Table 2 summarizes the results of phagocytosis mediated by FcγRI, FcγRII, and CD13, as well as phagocytosis of *E. coli* and zymosan, by non-polarized and the three types of polarized macrophages.

**Table 2:** **Summary of major findings in the different activation phenotypes**.

		MΦ-M0	MΦ-IFN-γ	MΦ-IL-4	MΦ-IL-10
Phagocytosis	FcγRI mediated	++++	+	+	+++++
	FcγRII mediated	++++	+	++	++++
	CD13 mediated	+++	+	+	++++
	*Escherichia coli*	++++	+	++++	+++++
	Zymosan	++	+	++++	++
ROS production	FcγRI mediated	+	+++++	0	+
	FcγRII mediated	+	+++++	0	+
	CD13 mediated	+	+++++	0	+
	FcγRI-/FcγRII mediated	+	+++++	0	+++
	FcγRI-/FcγRII-/CD13 mediated	++	+++++	0	+++

### Aggregation of FcγRI, FcγRII, or CD13 on Mϕ-IFN-γ Macrophages Induces ROS Production

**Figure 7:**
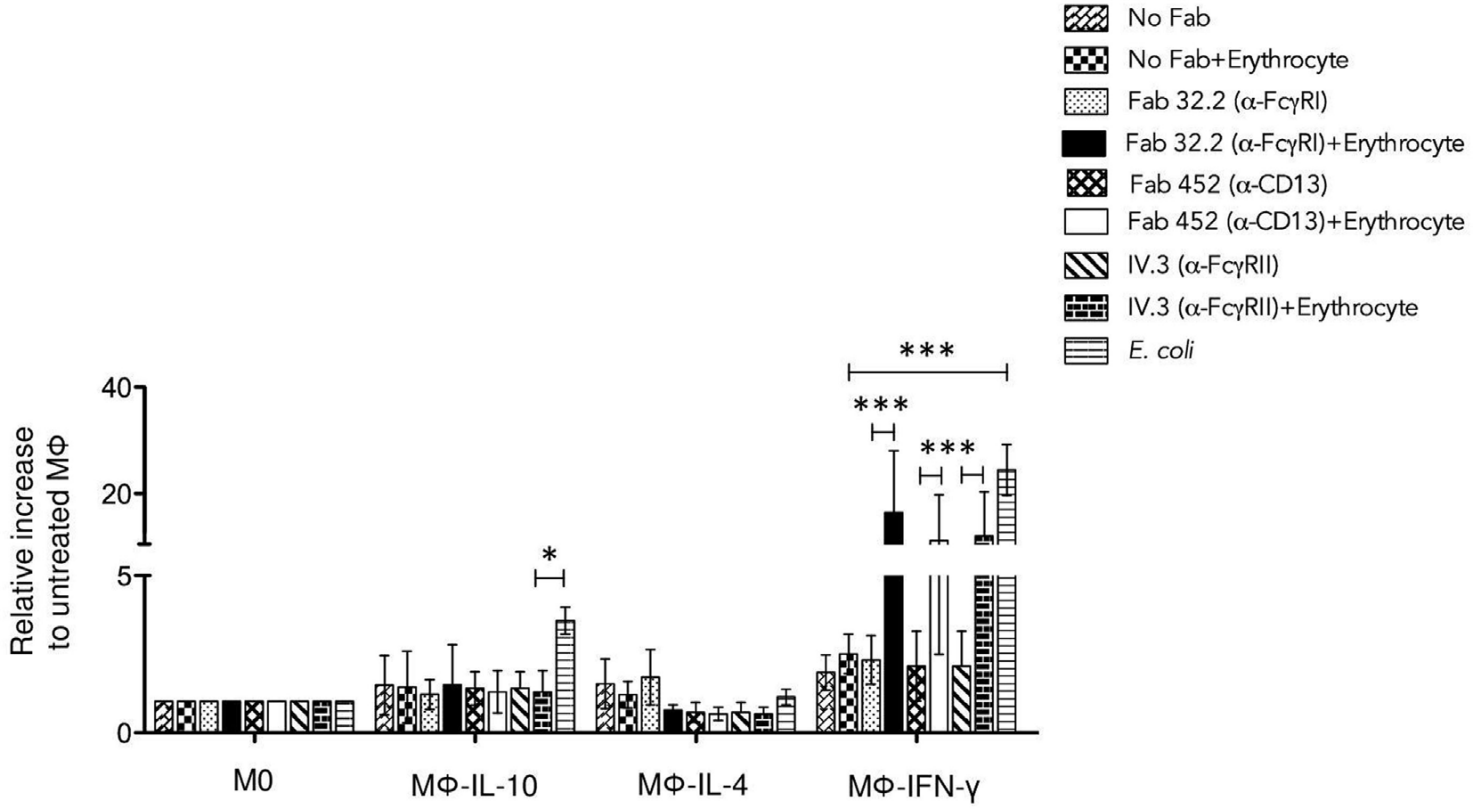
**Aggregation of FcγRI, FcγRII, or CD13 induces strong reactive oxygen species production in Mϕ-IFN-γ**. Non-polarized or polarized macrophages were incubated with 4 μg Fab fragments of mAb32.2 (Fab 32.2; anti-FcγRI) or Fab fragments of mAbIV.3 (Fab IV.3; anti-FcγRII) or 2 μg Fab fragments of mAb452 (Fab 452; anti-CD13) or without antibody (No Fab) for 30 min at 4°C. The cells were loaded with carboxy-H2DFFDA, for 30 min at 37°C. After washing, the receptors FcγRI, FcγRII, and CD13 were crosslinked or not for 30 min at 37°C by incubation with EBS-Fab. The cells were transferred to wells of black 96-well Inmuno Plates and the fluorescence from oxidized carboxy-H2DFFDA was determined after incubation for 30 min (total stimulation time: 60 min) as described in Section “Materials and Methods.” Data were normalized considering the value obtained for M0 macrophages as 1.0. Stimulation with *Escherichia coli* was used as a positive control. Results are expressed as mean ± SD of independent experiments. Statistical significance was calculated using one-way ANOVA with Tukey *post hoc* test (*p < 0.05, **p < 0.01, ***p < 0.001).

Macrophages are phagocytic cells that produce and release ROS in response to phagocytosis or stimulation with various agents. ROS generation has been implicated in a variety of physiological responses and is a critical component of the antimicrobial repertoire of macrophages. Therefore, we investigated whether FcγRI-, FcγRII-, or CD13-mediated phagocytosis results in generation of ROS in each population of macrophages. Cells were incubated on ice with Fab fragments of anti-FcγRI mAb32.2 (Fab 32.2) or Fab fragments of anti-FcγRII mAb IV.3 (Fab IV.3) or Fab fragments of anti-CD13 (Fab 452) or no treatment (No Fab). After this, cells were washed and loaded with carboxy-H2DFFDA (ROS-sensitive fluorescent dye). Cells were washed, and the receptors FcγRI, FcγRII, and CD13 were crosslinked for 30 min at 37°C by incubation with EBS-Fab (without CFSE), so as to replicate the stimulation leading to phagocytosis. Cells and EBS-Fabs were transferred to black 96-well plates, and the fluorescence of oxidized carboxy-H2DFFDA was measured at different times. As a control, macrophages were also stimulated with heat-killed *E. coli*. In M0 macrophages, phagocytic stimulation through FcγRI, FcγRII, and CD13 as well as *E. coli*, induced a slow ROS response that after 150 min became significantly higher compared to ROS production by cells treated only with EBS-Fabs. In contrast, Mϕ-IFN-γ produced significant amounts of ROS after only 30 min. Figure 7 shows ROS production by the different populations of polarized macrophages after 60 min of stimulation with different stimuli. To evaluate the effect of the polarization on ROS production, the results are shown relative to the ROS production induced by each stimulus in M0 cells. Compared to non-polarized macrophages, crosslinking of FcγRI, FcγRII, or CD13 induces significantly higher production of ROS in Mϕ-IFN-γ when crosslinking was induced by a phagocytable particle (EBS-Fab) (Figure 7) [Fab 32.2 (α-FcγRI) + EBS-Fabs, mean 16.4-fold increase] (*p* < 0.001); [Fab 452 (α-CD13) + EBS-Fab, mean 11.13-fold increase] (*p* < 0.01); (Fab IV.3 [α-FcγRII] + EBS-Fab, mean 11.9-fold increase) (*p* < 0.01). The increase in ROS generation by phagocytic stimuli through FcγRI, FcγRII, or CD13 was similar to the generation of ROS by stimulation with *E. coli* (positive control) (Figure 7) (*E. coli*, mean 24.4-fold increase) (*p* < 0.001). In contrast, Mϕ-IL-4 and Mϕ-IL-10 showed no difference in ROS production compared to M0 cells, after 60 min of stimulation by FcγRI, FcγRII, or CD13 crosslinking (Figure 7).

Altogether, these results suggest that phagocytosis through FcγRI, FcγRII, and CD13 in Mϕ-IFN-γ leads to strong generation of ROS that could potentially promote the degradation of ingested material.

### Mϕ-IL10 Are Able to Produce ROS after Coaggregation of FcγRI, FcγRII, and CD13

**Figure 8:**
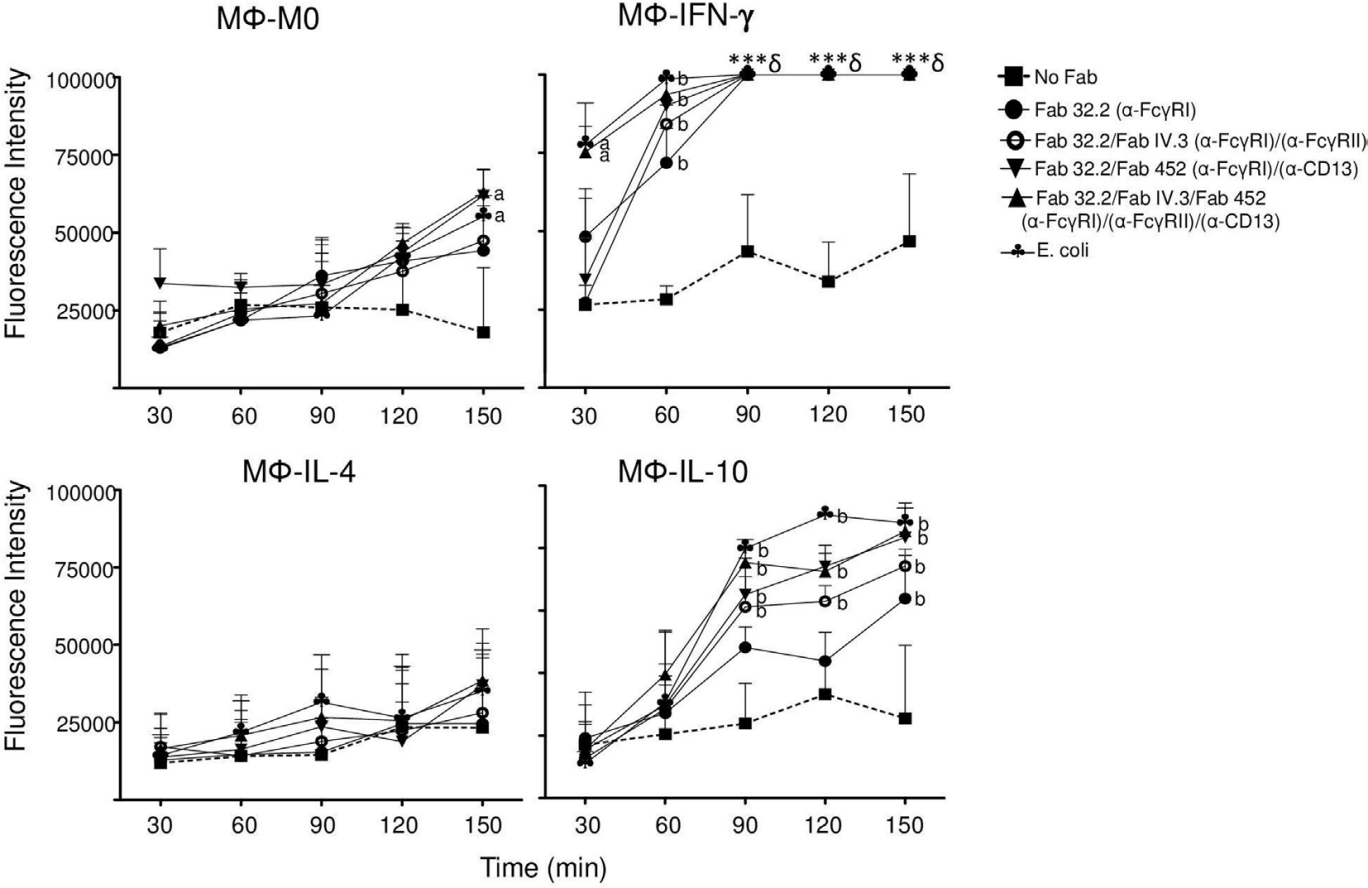
**Coaggregation of FcγRI, FcγRII, and CD13 induces reactive oxygen species production in Mϕ-IL-10**. Non-polarized or polarized macrophages were preincubated on ice with Fab fragments of anti-FcγRI (Fab 32.2) alone, or with Fab 32.2 + Fab fragments of anti-FcγRII (Fab IV.3), or with Fab fragments 32.2 + Fab fragments of anti-CD13 (Fab 452) or with three Fab fragments, 32.2 + IV.3 + 452, or no treatment (No Fab). The cells were loaded with carboxy-H2DFFDA, for 30 min at 37°C. After washing, the receptors were crosslinked for 30 min at 37°C by incubation with EBS-Fab. The cells were transferred to wells of black 96-well Inmuno Plates, and the fluorescence from oxidized carboxy-H2DFFDA was determined immediately and every 30 min as described in Section “Materials and Methods.” Results are expressed as mean ± SD of independent experiments (*n* = 15). Stimulation with *Escherichia coli* was used as a positive control. Statistical significance was calculated using one-way ANOVA with Tukey *post hoc* test. a = *p* < 0.01, b = *p* < 0.001 (***§ = Value overflowed the detection limit of the instrument).

We observed that although Mϕ-IL-10 did not produce significant amounts of ROS after crosslinking of FcγRI, FcγRII, or CD13, these cells did show a modest but significant ROS production after stimulation with *E. coli* (mean 3.5-fold increase relative to M0 macrophages, Figure 7). Although anti-inflammatory M2 macrophages are usually described as non-ROS-producing cells (36), it was recently reported that stimulation with PMA induces ROS production by M2 macrophages (37). Thus, we considered the possibility that Mϕ-IL-10 could produce ROS, albeit at levels lower than those produced by Mϕ-IFN-γ, and this led us to evaluate ROS production at longer times and under stimulation by two or more receptors. For this, different subpopulation of macrophages were preincubated on ice with Fab fragments of anti-FcγRI mAb32.2 (Fab 32.2) alone, or with Fab 32.2 and Fab fragments of anti-FcγRII mAb IV.3 (Fab IV.3), or with Fab fragments 32.2 and Fab fragments of anti-CD13 (Fab 452) or with the three Fab fragments (32.2, IV.3, and 452), or no treatment (No Fab). Subsequently, cells were washed and loaded with carboxy-H2DFFDA. Cells were washed and incubated for 30 min at 37°C with EBS-Fab (without CFSE), so as stimulate phagocytosis. Cells and EBS-Fabs were transferred to black 96-well plates, and the fluorescence of oxidized carboxy-H2DFFDA was read immediately (initial reading) and for 2 h 30 min, with reading intervals every 30 min. As a positive control, macrophages were also stimulated with heat-killed *E. coli*. The results are shown in Figure 8. Mϕ-IFN-γ generated high levels of ROS after stimulation through FcγRs and CD13 or by *E. coli*, such that the fluorescence signal overflowed the detection limit of the instrument after 60 min. Mϕ-IL-10, on the other hand, produced ROS at amounts that became significant after 90 min of stimulation by *E. coli* or by coaggregation of two (FcγRI and FcγRII or FcγRI and CD13) or three (FcγRI + FcγRII + CD13) receptors. ROS production by crosslinking of FcγRI alone was significant only after 150 min (Figure 8), while crosslinking FcγRII or CD13 separately did not induce significant production of ROS (data not shown). The increase in ROS generation by stimulation of Mϕ-IL-10 simultaneously through more than one phagocytic receptor was similar to the generation of ROS by stimulation with *E. coli* (Figure 8). In contrast, Mϕ-IL-4 did not produce significant levels of ROS under any stimulation even after 150 min. These results suggest that simultaneous stimulation through Fcγ receptors and CD13 as well as stimulation with *E. coli* are strong signals that are able to induce ROS production in Mϕ-IL-10 macrophages, although this production is significantly lower than ROS generation in Mϕ-IFN-γ.

Table 2 summarizes the results of ROS production by non-polarized and the three types of polarized macrophages after stimulation through FcγRI, FcγRII, and CD13 separately and in combination.

## Discussion

Macrophages are very plastic cells that can acquire distinct phenotypes (activation states) under the influence of different cytokine microenvironments. Although several authors have shown that macrophages treated with different stimuli display altered phenotypes or functional capacities, many of these studies are limited in that they compare only one particular activation state with non-polarized macrophages. To complicate matters more, different laboratories have used different activation protocols on different human or murine cell populations, making it difficult to perform direct comparisons among studies [discussed in Murray et al. (4), although centered on the murine system but of equal relevance for studies of human cells]. Although macrophage activation was initially seen as a dichotomy between classically and alternatively activated macrophages, it is now clear that the spectrum of macrophage activation states is much more diverse (38, 39). Thus, studies aimed at comparing macrophage activation phenotypes under standardized conditions are necessary to create a framework that could serve as a reference for *in vivo* studies of macrophage activation.

In this study, we have compared three populations of activated (polarized) macrophages, which have been proposed as representative of different phenotypes that could be related to three important functions of macrophages: inflammation and microbial killing, wound repair, and immune regulation (40). The different activated populations were induced by the treatment of hMDMs with three archetypical cytokines: IFN-γ, IL-4, and IL-10. In much of the macrophage literature, the populations resulting from treatment with these cytokines have been referred to as M1, M2a, and M2c macrophages, although such designations have been also used for macrophages polarized under different stimulation protocols. We follow the suggestions of Murray et al (4) and refer to these populations as Mϕ-IFN-γ, Mϕ-IL-4, and Mϕ-IL-10, as these designations are unambiguous, and refer to non-polarized macrophages as M0.

Our first aim was to define specific markers that could be used to characterize these distinct polarized macrophage subsets. Identification of specific phenotypic markers is important because it provides an objective definition of the activation state of the cells that we used. Moreover, the activation phenotypes could be used as a reference for delineation of the activation phenotype of macrophages found *in vivo* under different physiological or pathological conditions or *in vitro* after different activation protocols. Also, as more investigators use the same markers to characterize and/or define the activation state of the cell population studied, this will facilitate unambiguous interpretation of results from different groups.

Our results identified CD80, CD86, and CD64^high^ as markers for Mϕ-IFN-γ, expression of CD209, upregulation of CD206 and CD11b and downregulation of CD14 for Mϕ-IL-4, and expression of CD163, CD64, and CD16 for Mϕ-IL-10. Ambarus et al. (32) previously reported a study to validate specific markers of human monocytes cultured with IFN-γ, IL-4, and IL-10. They proposed CD80 as the best phenotypic marker for human Mϕ-IFN-γ, upregulation of CD200R and down regulation of CD14 as distinctive of Mϕ-IL-4, and expression of CD163 and CD16 as specific markers for Mϕ-IL-10. Some experimental differences exist between their study and ours. First, they started cytokine treatment on freshly isolated monocytes, while we first incubated the isolated monocytes for 6 days with M-CSF (5 ng/mL), to perform cytokine treatment on fully differentiated macrophages. Although monocyte treatment with M-CSF has been used to polarize macrophages to an alternative phenotype (41), the concentration we used (5 ng/mL) is at least an order of magnitude lower than the ones used in those studies (50–100 ng/mL), and we found no significant differences in expression of CD206 between cells cultivated with or without M-CSF. We decided to include M-CSF during monocyte-to-macrophage differentiation because we noticed that M-CSF treatment greatly improved cell viability, and the response to cytokines showed less variability among cells from different donors. Despite these differences, in general terms, both studies show good agreement, identifying CD80 and CD64^high^ as markers of Mϕ-IFN-γ, significant expression of CD206 and down regulation of CD14 as characteristics of Mϕ-IL-4, and expression of CD163 and CD16 as markers for Mϕ-IL-10 [(32) Figures 1 and 2]. Both studies observed a significant increase in expression of CD86 on Mϕ-IFN-γ, but since this marker has been variably found to increase also after IL-4 treatment (32, 42), we consider it unsuitable as a specific marker of Mϕ-IFN-γ. With respect to Mϕ-IL-4, we found a significant increase in CD209 (DC-SIGN) and consider it suitable as a characteristic marker of this population of polarized macrophages. CD209 has been previously reported to be specifically expressed by IL-4 treated macrophages (43, 44) and has been proposed as a marker for alternatively activated macrophages (45).

Along with the distinctive expression of membrane markers, we observed different patterns of cytokine secretion among the three different subpopulations of macrophages. Non-polarized M0 macrophages did not secret significant amounts of any of the cytokines tested, neither without stimulation nor after stimulation with LPS. Basal (unstimulated) cytokine production by Mϕ-IFN-γ was characterized by TNF-α, IL-8, and IL-6 secretion (Figure 4), although we found statistical difference only for secretion of TNF-α (*p* < 0.05). Mϕ-IL-10 showed basal expression of low levels of IL-10 (*p* < 0.05) (Figure 3). Upon stimulation with LPS, Mϕ-IFN-γ secreted IL-8, IL-6, IL-1β, TNF-α, and IL-12p70, with IL-8 being the most abundantly secreted cytokine. Both Mϕ-IL-4 and Mϕ-IL-10 stimulated with LPS secreted significant levels of IL-10 (*p* < 0.001). In summary, Mϕ-IFN-γ macrophages, which express higher levels of costimulatory molecules, preferentially secrete pro-inflammatory cytokines suggesting that these cells are capable of responding efficiently to microbial/endotoxin challenges (39), whereas Mϕ-IL-4 and Mϕ-IL-10 macrophages produce the anti-inflammatory cytokine IL-10. Although IL-10 is a hallmark M2 cytokine in the mouse (40), we and others (41) have shown that in humans, Mϕ-IFN-γ macrophages stimulated with LPS secrete low but significant (compared to non-polarized macrophages) levels of IL-10 (*p* < 0.05) (Figure 4). Thus, based on the membrane markers expressed and the cytokines produced, it is evident that human Mϕ-IFN-γ have several characteristics of what is usually considered as M1 pro-inflammatory macrophages, Mϕ-IL-4 have characteristics of M2a (tissue-repairing) macrophages, and Mϕ-IL-10 those of M2c (regulatory) macrophages.

Since we were interested in evaluating FcγR- and CD13-mediated phagocytosis in the polarized macrophages, we determined the effect of polarization on the expression of these receptors. We observed that CD64 (FcγRI) was significantly upregulated by IFN-γ (Figure 2), which agrees with previous reports (31, 32). IL-10 also induced an increase in CD64 expression compared to the non-polarized and IL-4-treated macrophages, although this increase was significantly smaller than the increase induced by IFN-γ (Figure 2). Ambarus and colleagues (32) also observed a small increase in CD64 expression in Mϕ-IL10 compared to M0 and Mϕ-IL-4 cells, although in their experiments, this increase was not statistically significant. IL-10 induced also the expression of FcγRIII (CD16). The membrane expression of CD32 (FcγRII), as well as the expression of CD13, did not change after treatment with any of the polarizing cytokines. Monocytic cells can express two CD32 isoforms (FcγRIIa and FcγRIIb). Although their extracellular domains are very similar, the receptors have opposite functional activities: FcγRIIa is an activating receptor that contains an intracellular ITAM, while FcγRIIb isoform is an inhibitory receptor that contains an ITIM in its cytoplasmic portion (46). Thus, since changes in the relative expression of the two isoforms could affect functions mediated by FcγRII, we analyzed by qRT-PCR the effect of the different polarizing treatments on the expression of mRNA for FcγRI, FcγRIIa, FcγRIIb, FcγRIII, and CD13. The results show that although expression of CD32 on the membrane was not changed after polarization, the ratios FcγRIIa/FcγRIIb of activatory/inhibitory isoforms of the receptor were distinctly modulated. With respect to the ratio in non-polarized cells, the ratio is higher in Mϕ-IL-10 and lower in Mϕ-IL-4 and Mϕ-IFN-γ and probably contributes to the higher phagocytosis displayed by Mϕ-IL-10, both of IgG-opsonized erythrocytes as well as in selective phagocytosis through FcγRII. Significant increases in mRNA for FcγRI were observed in Mϕ-IFN-γ and Mϕ-IL-10, and in mRNA for FcγRIII in Mϕ-IL-10, which are reflected in the membrane expression of these receptors.

By using a phagocytosis assay that allowed us to target labeled SRBCs to specific receptors on the cell surface, we were able to analyze phagocytosis mediated specifically by FcγRI, FcγRII, or CD13. We have recently reported that CD13 is a phagocytic receptor in human monocytic cells (14). As shown in Figures 4 and 5, phagocytosis through each of these receptors follows a similar pattern in the different macrophage populations: Mϕ-IFN-γ showed the lowest phagocytic activity, while Mϕ-IL-10 were highly phagocytic. M0 cells were also capable of significant phagocytosis through the three receptors, only slightly less than Mϕ-IL-10. Although IFN-γ induces a significant increase in FcγRI expression, phagocytosis through this receptor is significantly lower in Mϕ-IFN-γ. In contrast, Mϕ-IL-10, which showed a smaller increase in FcγRI expression, showed a significantly higher FcγRI-mediated phagocytosis (Figure 4A). Since Mϕ-IL-10 showed the highest phagocytosis also through FcγRII and CD13, which did not show changes in expression, this suggests that the phagocytic activity is more dependent on cellular properties/characteristics other than expression of the corresponding receptors on the cell membrane. In line with this suggestion, Mϕ-IL-4, whose expression of FcγRI, FcγRII, and CD13 on the cell membrane was not different from M0 untreated macrophages, showed very low levels of phagocytosis through these receptors.

The significantly lower ability of Mϕ-IFN-γ to phagocytose through FcγRs and CD13 is also evident in phagocytosis of heat-killed *E. coli* and zymosan, which are internalized through different receptors (Figure 6). In contrast, both non-polarized and Mϕ-IL-10 internalize both particles very efficiently. However, it is not possible to generalize and consider a particular activation phenotype as highly or poorly phagocytic, since while Mϕ-IL-4 are very poorly phagocytic through FcγRs and CD13, these cells are as efficient as Mϕ-IL-10 for phagocytosis of *E. coli* and are the population with the highest ability for phagocytosis of zymosan particles (Figure 6).

Our results and those of others (19, 47, 48) have shown that in human monocytes/macrophages, IL-10 increases phagocytosis of *E. coli* (Figure 6A) and IgG- opsonized particles (Figure S2 in Supplementary Material), suggesting that the effects of IL-10 are not specific for a particular phagocytic receptor. It is possible that, in addition to increasing the expression of phagocytic receptors (CD64, CD32, CD16, and scavenger receptor CD163), IL-10 modulates the expression and/or activation of signaling molecules involved in phagocytic pathways, resulting in enhanced phagocytosis through these receptors, which is one of the features of M2c macrophages. IL-10 drives macrophages toward a phenotype involved in tissue repair and dampening of inflammation (47, 48).

To date, the mechanisms by which IL-10 enhances and IFN-γ downregulates phagocytosis are not known. Previous studies from our group investigated the effect of IFN-γ and IL-10 on phagocytic signaling mediated by FcγRs in monocytic cells (19). In that study, it was shown that the inhibition of phagocytosis induced by IFN-γ was not accompanied by inhibition of the early biochemical events triggered by FcγRs crosslinking. It was found that IFN-γ induced a higher basal level of F-actin and activation of Rac1, which might be involved in the reduced phagocytic capacity of IFN-γ-treated cells. Inhibition of PI3K prevented the increase in F-actin induced by IFN-γ. Thus, it was proposed that F-actin assembly in response to IFN-γ engages a portion of the components necessary for cytoskeleton reorganization, leading to impaired responses requiring cytoskeleton rearrangement. Previous reports have demonstrated that IFN-γ can diminish monocyte responses that depend on cytoskeleton reorganization. Thus, monocytes cultured in the presence of IFN-γ showed defective internalization of bacteria and SRBC, unopsonized or opsonized with C3b/iC3b or IgG (34, 49–51). Here, we also found that treatment with IFN-γ inhibits phagocytosis of *E. coli* and IgG-opsonized particles compared with M0, Mϕ-IL-4, and Mϕ-IL-10 (Figure 6A).

Phagocytes produce ROS conjunctly with phagocytosis or after stimulation with various agents. ROS generation has been implicated in a variety of physiological responses and is a critical component of the antimicrobial repertoire of phagocytes. Thus, we investigated whether FcγRI, FcγRII, and CD13-mediated phagocytosis is coupled to ROS production and if this production is regulated by the activation phenotype of the cell. Uptake of antigens *via* activating Fcγ receptors (mainly FcγRI and FcγRII) has been reported to result in enhanced NADPH oxidase assembly and production of ROS (52). On the other hand, IL-4 induces downregulation of the gp91 subunit of the NOX2 complex (53), which results in lower production of ROS but increases the reducing capacity of the phagosome. Our results show a difference in ROS production among the different activation phenotypes. Mϕ-IFN-γ macrophages have a higher ROS-producing capacity compared to M0, Mϕ-IL-4, and Mϕ-IL-10 macrophages (Figure 8). Furthermore, we found that ROS production was induced only when the receptors were crosslinked by a phagocytable particle, suggesting that ROS production is specifically linked to the phagocytic process. As expected, our results indicate that FcγRI, FcγRII, and CD13-mediated phagocytosis induces ROS production in Mϕ-IFN-γ macrophages and not in Mϕ-IL-4 and Mϕ-IL-10 (Figure 8). Traditionally, ROS-producing capacity has been associated with pro-inflammatory macrophages, but less with anti-inflammatory macrophages. This is consistent with our results. Nevertheless, Kraaij and colleagues (37, 54) showed that upon PMA stimulation, M2 subsets have different ROS-producing capacity: M2c macrophages have a higher ROS production compared to M2a macrophages, which is most likely related to a reduced expression of gp91^phox^ by the later. We investigated if Mϕ-IL-10 could produce ROS by simultaneous stimulation of several phagocytic receptors and found that, while in Mϕ-IFN-γ coaggregation of FcγRI/FcγRII/CD13 induces a high production of ROS from the first 30 min of stimulation (Figure 8), simultaneous stimulation of phagocytic receptors in Mϕ-IL-10 does not induce significant production of ROS at the same time (30 and 60 min). However, after 90 min of simultaneous stimulation through phagocytic receptors or stimulation by *E. coli*, ROS production by Mϕ-IL-10 macrophages is significantly higher than in unstimulated cells (Figure 8), and the production increases with respect to time. In contrast, simultaneous stimulation of several receptors does not induce ROS generation by Mϕ-IL-4 macrophages at any time analyzed. In summary, Mϕ-IFN-γ and Mϕ-IL-10 macrophages produced significantly higher quantities of intracellular ROS compared to M0 and Mϕ-IL-4 macrophages. The high levels of ROS production by M1 macrophages has been previously described and is in line with their pro-inflammatory role. To our knowledge, ROS production associated with phagocytosis in Mϕ-IL-10 macrophages has not been reported previously.

The mechanisms and functional relevance of ROS production by M2 macrophages has only started to be investigated. Kraaij and colleagues (54) compared the expression of two proteins (p47 and gp91) of the NOX2 complex in M2a and M2c macrophages. Both mRNA and protein expression of p47^phox^ was similar between M2 subsets, but the mRNA and protein expression of gp91^phox^ were lower in M2a compared with M2c macrophages, which correlates with the higher production of ROS by M2c macrophages. Although ROS production is mainly considered to be pro-inflammatory, causing cell and tissue destruction, recent findings have shown that ROS is also involved in regulation of immune responses as they can have an anti-inflammatory role and prevent autoimmune responses (55). In this respect, it is noteworthy that human M2 macrophages have been shown to suppress T cell responses by induction of T regulatory cells (Tregs) in a ROS-dependent manner (56). Tregs play a critical role in the prevention of autoimmunity and resolution of inflammation. Interestingly, the immunosuppressive drug dexamethasone was shown to increase the ability of M2 macrophages to produce ROS, and injection of dexamethasone in rats caused a long-lasting upregulation of ROS production by macrophages and induced higher levels of Tregs in a ROS-dependent manner (57).

Throughout this study, we compared the phenotype and functions of polarized macrophages with those of non-polarized macrophages. M0 macrophages could represent a macrophage in its “basal” state, probably related to cells resident in tissues in homeostasis, in the absence of microbial or cytokine stimulation. However, these cells are by no means dormant or quiescent. They are actively engaged in maintaining tissue homeostasis and are expected to be capable of phagocytosis of apoptotic cells as well as bacteria and particulate matter. It is thus no surprising that M0 cells showed efficient phagocytosis of all particles tested and even a small and late ROS production. In this respect, polarization could be considered more an increment or dampening of functions already present in the non-polarized macrophage, rather than inducing the appearance of entirely new capacities in the cell.

In conclusion, we have analyzed the expression and the ability to mediate effector functions of the phagocytic receptors FcγRs and CD13, in three populations of *in vitro* polarized human macrophages and non-polarized macrophages. We characterized these populations in terms of membrane markers and cytokine production. We found that the ability to mediate phagocytosis and ROS production depend more heavily on the activation state of the macrophage than on the expression level of the specific receptor. Thus, phenotypic and transcriptomic profiling of differently activated cells could serve as a guide for their ability to perform specific functions, but it is essential to carry on functional experiments. Along these lines, caution should be exerted when considering a specific macrophage population with generalizations such as “highly phagocytic,” as exemplified by Mϕ-IL-4, which are highly phagocytic of zymosan particles, but show very low FcγR-mediated phagocytosis despite significant expression of the later receptors.

## Author Contributions

EM-C and EO conceived and designed the experiments, analyzed the results, and wrote the manuscript. EM-C performed the experiments and wrote the first draft of the manuscript.

## Conflict of Interest Statement

The authors declare that the research was conducted in the absence of any commercial or financial relationships that could be construed as a potential conflict of interest.

## Abbreviations

ATCC, American Type Culture Collection; Carboxy-H2DFFDA, 5-(and-6)-carboxy-2′,7′-difluorodihydrofluorescein diacetate; CD13, cluster of differentiation 13, aminopeptidase N; CFSE, carboxyfluorescein succinimidyl ester; EBS-Fab, sheep red blood cells labeled with CFSE and coated with biotin, streptavidin, and biotin-labeled bivalent antigen-binding fragments of anti-IgG; F(ab′)2, bivalent antigen-binding fragments of antibodies (disulfide bond joined); FcγRs, receptors for the Fc portion of immunoglobulin G; FcγRI, type 1 high-affinity receptor for the Fc portion of immunoglobulin G (CD64); FcγRII, type II receptor for the Fc portion of immunoglobulin G (CD32); FcγRIII, type III receptor for the Fc portion of immunoglobulin G (CD16); Mϕ, macrophage; hMDM, human monocyte-derived macrophage; Mϕ-IFN-γ, IFN-γ-treated macrophages; Mϕ-IL-4, IL-4-treated macrophages; Mϕ-IL-10, IL-10-treated macrophages; M-CSF, macrophage colony-stimulating factor; PI, phagocytic index; ROS, reactive oxygen species; SRBC, sheep red blood cells.
