# Supplementary material for: Macrophage Polarization Modulates FcγR- and CD13-Mediated Phagocytosis and Reactive Oxygen Species Production, Independently of Receptor Membrane Expression
Source: Front Immunol. 2017 Mar 27;8:303. doi: 10.3389/fimmu.2017.00303 (PMC5366847; doi:10.3389/fimmu.2017.00303)
Supplement: Supplementary file 1 [file Image_1.PDF]

SUPPLEMENTARY FIGURE 1

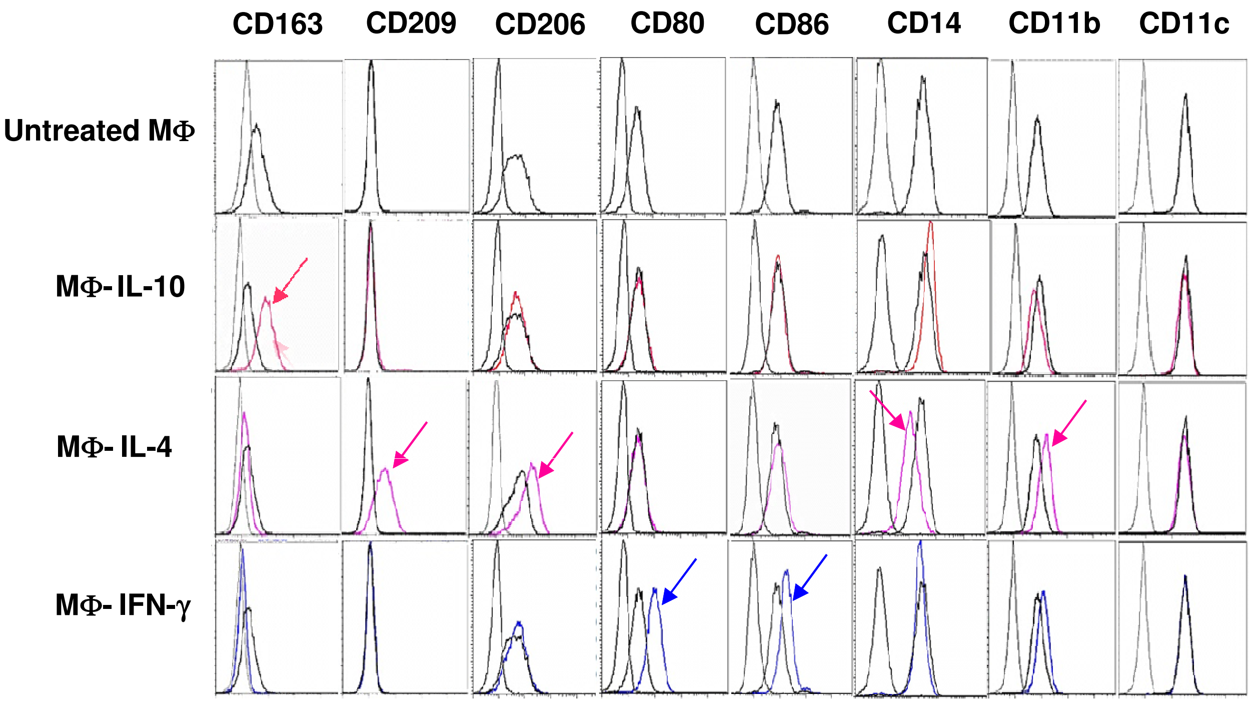

Supplementary Figure 1. Cell surface markers expressed on *in vitro* polarized macrophages. Monocytes from peripheral blood of healthy donors were cultured for 6 days in medium containing M-CSF to differentiate into M0. The resulting hMDM were polarized by incubation with IFN-γ (30 ng/mL), IL-4 (50 ng/mL) or IL-10 (20 ng/mL) for 48 hrs and subsequently analyzed by flow cytometry for the expression of surface markers. Representative histograms of cells from a single donor (out of 30). Arrows indicate significant changes in expression induced by polarization.
