# Supplementary material for: Macrophage Polarization Modulates FcγR- and CD13-Mediated Phagocytosis and Reactive Oxygen Species Production, Independently of Receptor Membrane Expression
Source: Front Immunol. 2017 Mar 27;8:303. doi: 10.3389/fimmu.2017.00303 (PMC5366847; doi:10.3389/fimmu.2017.00303)
Supplement: Supplementary file 2 [file Image_2.PDF]

SUPPLEMENTARY FIGURE 2

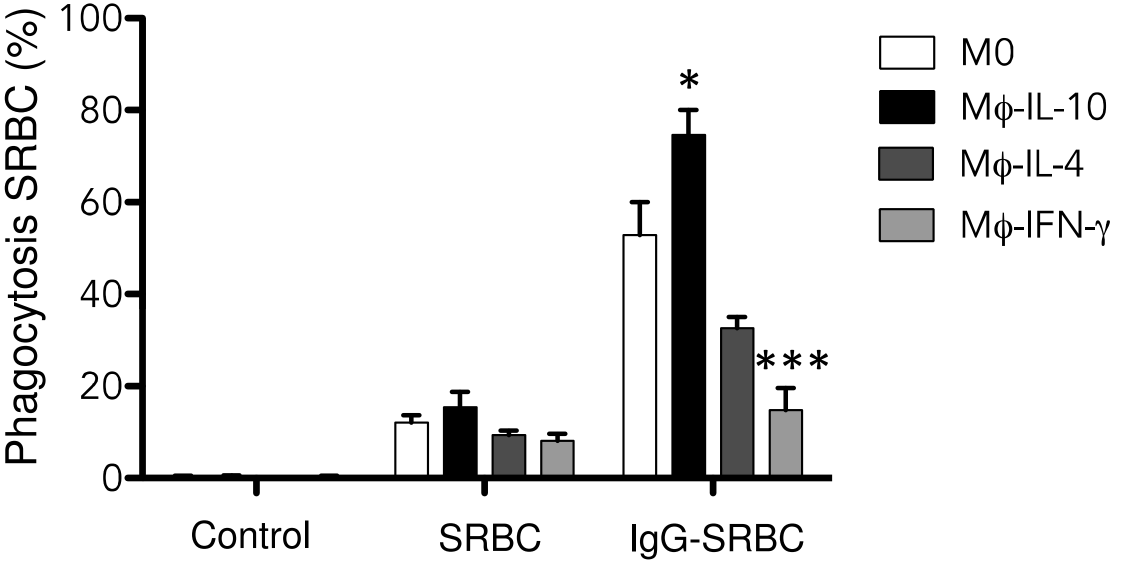

Supplementary Figure 2. Interferon-γ inhibits phagocytosis of IgG-SRBC. M0 macrophages were polarized with IFN-γ, IL-4 or IL-10. After 48 h. MDM were harvested and incubated with IgG-SRBC for 30 min at 37ºC. Non-internalized SRBC were lysed, and phagocytosis was evaluated by flow cytometric. Percentages of phagocytosis were calculated relative to control (non-IgG-SRBC). Results are expressed as mean + SEM of ten independent experiments. Statistical significance was calculated using nonparametric one-way ANOVA with Tukey *post hoc* test. *p <0.05, **p <0.01, ***p < 0.001.
